# Mechanistic Studies on Dehydration in Class V Lanthipeptides

**DOI:** 10.1021/acschembio.2c00458

**Published:** 2022-08-31

**Authors:** Haoqian Liang, Isaiah J. Lopez, Marina Sánchez-Hidalgo, Olga Genilloud, Wilfred A. van der Donk

**Affiliations:** † Department of Biochemistry, University of Illinois at Urbana—Champaign, 600 S. Mathews Avenue, Urbana, Illinois 61801, United States; ‡ Department of Chemistry and Howard Hughes Medical Institute, University of Illinois at Urbana—Champaign, 600 S. Mathews Avenue, Urbana, Illinois 61801, United States; § Fundación MEDINA Centro de Excelencia en Investigación de Medicamentos Innovadores en Andalucía, Avenida del Conocimiento, 34 Parque Tecnológico de Ciencias de la Salud, Armilla, 18016 Granada, Spain

## Abstract

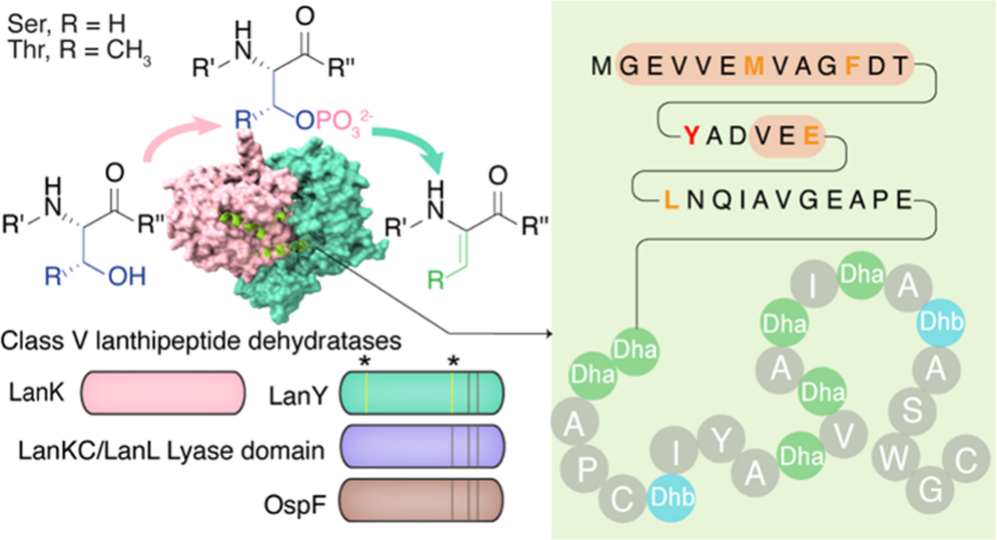

Lanthipeptides are ribosomally synthesized and post-translationally modified peptides characterized by lanthionine (Lan) and/or methyllanthionine (MeLan) residues. Four classes of enzymes have been identified to install these structures in a substrate peptide. Recently, a novel class of lanthipeptides was discovered that lack genes for known class I–IV lanthionine synthases in their biosynthetic gene cluster (BGC). In this study, the dehydration of Ser/Thr during the biosynthesis of the class V lanthipeptide cacaoidin was reconstituted *in vitro*. The aminoglycoside phosphotransferase-like enzyme CaoK iteratively phosphorylates Ser/Thr residues on the precursor peptide CaoA, followed by phosphate elimination catalyzed by the HopA1 effector-like protein CaoY to achieve eight successive dehydrations. CaoY shows sequence similarity to the OspF family proteins and the lyase domains of class III/IV lanthionine synthetases, and mutagenesis studies identified residues that are critical for catalysis. An AlphaFold prediction of the structure of the dehydration enzyme complex engaged with its substrate suggests the importance of hydrophobic interactions between the CaoA leader peptide and CaoK in enzyme–substrate recognition. This model is supported by site-directed mutagenesis studies.

## Introduction

Ribosomally synthesized and post-translationally modified peptides (RiPPs) are a rapidly expanding class of natural products.^1^ The largest class of known RiPPs is the lanthipeptides, which are characterized by the β-thioether cross-linked bis amino acids lanthionine (Lan) and methyllanthionine (MeLan). Installation of (Me)Lan is achieved through dehydration of Ser/Thr residues to form dehydroalanine (Dha)/dehydrobutyrine (Dhb), followed by intramolecular Michael-type addition of cysteine thiols to the resulting dehydroamino acids.^2^ Four classes of enzymes have been characterized that differ in their domain architecture and mechanisms of (Me)Lan synthesis.^2^ Biosynthesis of class I lanthipeptides involves dedicated dehydratase (LanB) and cyclase (LanC) enzymes. LanB enzymes use glutamyl-tRNA to glutamylate the side chains of Ser/Thr residues, followed by glutamate elimination to generate Dha/Dhb. LanC enzymes use a zinc ion to activate the Cys thiol for addition to Dha/Dhb. In contrast, class II–IV lanthipeptides are formed by the multifunctional enzymes LanM (class II), LanKC (class III), and LanL (class IV) that catalyze both dehydration and cyclization. These three enzyme classes use phosphorylation by kinase domains to activate the side chain hydroxy groups of Ser/Thr for elimination. They differ in how they catalyze the elimination, which occurs within the kinase active site for LanM and in dedicated phosphoSer/phosphoThr lyase domains for LanKC and LanL. LanKC and LanL differ in their cyclase domains, which contain zinc in a LanC fold for LanL but takes place in a domain that lacks the zinc-binding site for LanKC. Recently, a novel group of (Me)Lan-containing RiPPs were discovered for which the biosynthetic gene cluster (BGC) lacked genes encoding well-defined class I–IV (Me)Lan synthase homologues, suggesting the existence of an unknown synthase.^3−6^ This new group of lanthipeptides was termed class V.^5^

**Figure 1:**
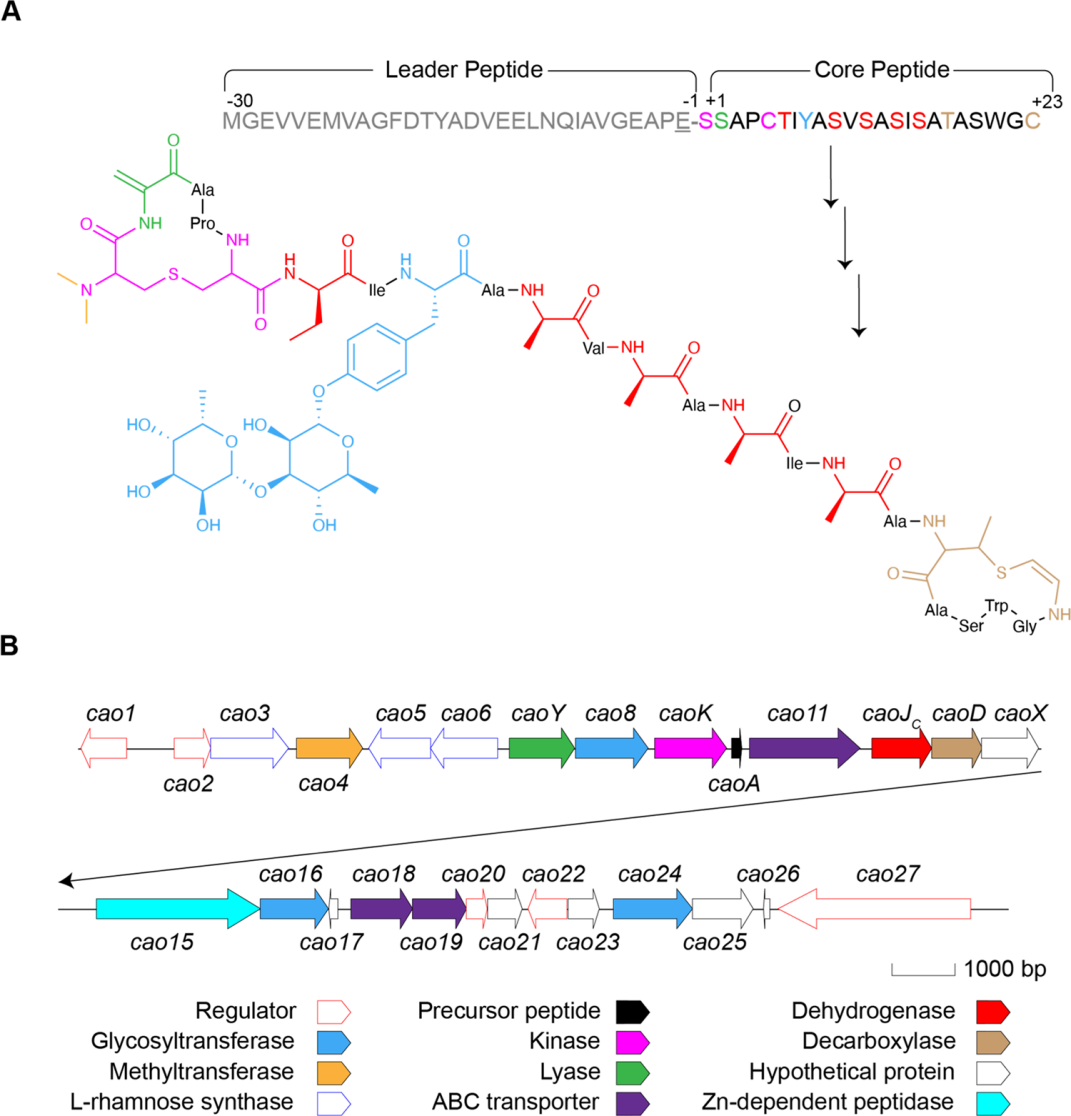
(A) Sequence of CaoA and structure of cacaoidin. The sequence of the CaoA core and leader peptides is depicted, as well as the residues that undergo post-translational modification. The cleavage sequence at the end of the leader peptide is shown in underlined font. In cacaoidin, the N-terminal Lan is depicted in pink, with di-methylation in orange. The C-terminal AviMeCys motif is depicted in brown. Dehydroalanine is shown in green, dehydroamino acids that are further reduced to d-Ala and d-Abu are shown in red, and glycosylated Tyr is shown in blue. (B) Biosynthetic gene cluster of cacaoidin.

Cacaoidin was the first reported class V lanthipeptide and is produced by *Streptomyces cacaoi* CA-170360 (Figure 1).^3,6^ Cacaoidin displays potent antimicrobial activity against Gram-positive bacteria, including methicillin-resistant *Staphylococcus aureus* (MRSA), and carries multiple unusual structural features, including an *N,N-*dimethyl lanthionine, d-amino acids, an *O-*glycosylated tyrosine, and a C-terminal aminovinyl-methyl-cysteine (AviMeCys) (Figure 1A). The *cao* BGC contains roughly 27 open reading frames (ORFs) (Figure 1B), with basic local alignment search tool (BLAST) analysis used to tentatively assign functions based on the modifications found in cacaoidin. Like for other lanthipeptides, the precursor peptide for cacaoidin is made up of an N-terminal leader peptide and a C-terminal core peptide (Figure 1A). The latter is converted into mature cacaoidin by post-translational modifications.^3,6^ A subset of proteins encoded in the *cao* BGC exhibit similarity with those in the BGCs of other recently reported class V lanthipeptides, the *lxm* BGC involved in lexapeptide biosynthesis^4^ and the *spr* BGC involved in pristinin A3 biosynthesis.^5^ These include a flavin-dependent cysteine decarboxylase (CaoD, formerly Cao13) for AviMeCys formation, an F_420_H_2_-dependent dehydrogenase (CaoJ_C_, formerly Cao12) involved in d-amino acid formation,^4^ a HopA1-like effector protein (CaoY, formerly Cao7), and two aminoglycoside phosphotransferase (APH) family proteins (CaoK and CaoX, formerly Cao9 and Cao14). The HopA1 and APH proteins are believed to be involved in dehydration and cyclization in class V lanthipeptides,^3−7^ but the *in vitro* activity has not yet been reported.

In this study, we demonstrate the dehydration of CaoA by the combined action of the APH protein CaoK and the HopA1 homologue CaoY. We also provide insight into the residues required for the activity of the lyase CaoY through predicted secondary structure analysis as well as mutagenesis. Following the nomenclature used for lexapeptide,^4^ we propose the use of LanK for the class V Ser/Thr kinases and LanY for the phosphoSer/phosphoThr lyases that together achieve dehydration to continue using common names in lanthipeptide biosynthesis.^8^ We also provide a model for the substrate recognition mechanism by the dehydratase complex formed by CaoK and CaoY using AlphaFold-Multimer structure prediction-guided site-directed mutagenesis.^9−11^

## Results and Discussion

### Dehydration of CaoA

**Figure 2:**
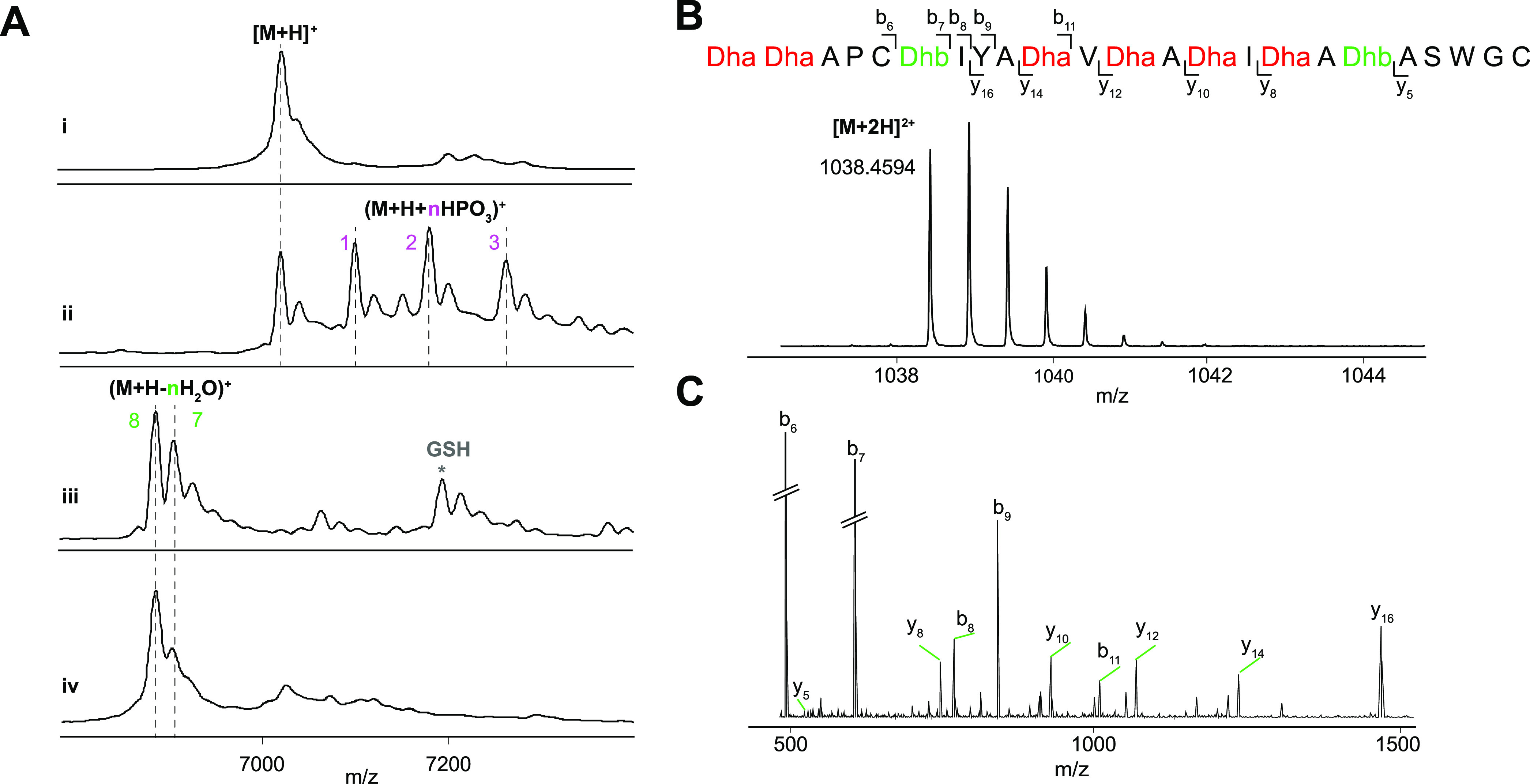
(A) MALDI-TOF mass spectra of (i) unmodified His_6_–CaoA. [M + H]^+^ exp. *m*/*z* = 7025; obsd *m*/*z* = 7022. (ii) His_6_–CaoA modified by CaoK in *E. coli*. [M + H]^+^ exp. = 7025, obsd = 7028; (M + H + HPO_3_)^+^ exp. = 7105, obsd = 7109; (M + H + 2HPO_3_)^+^ exp. = 7185, obsd = 7188; (M + H + 3HPO_3_)^+^ exp. = 7265, obsd = 7271; (M + H + 4HPO_3_)^+^ exp. = 7345, obsd = 7349. (iii) His_6_–CaoA modified by CaoK and CaoY in *E. coli*. (M + H – 8H_2_O)^+^ exp. = 6881, obsd = 6883; (M + H – 8H_2_O + GSH)^+^ exp. = 7186, obsd = 7190. (iv) His_6_–CaoA modified by His_6_–CaoK–CaoY dehydratase complex *in vitro* with adenosine triphosphate (ATP) and MgCl_2_. (M + H – 8H_2_O)^+^ exp. = 6881, obsd = 6878. (B, C) Liquid chromatography coupled with electrospray ionization-quadrupole-time of flight (LC-ESI-QTOF) mass spectrum of (B) Glu-C digested His_6_–CaoA core peptide modified by CaoK–CaoY during co-expression in *E. coli* ([M + 2H]^2+^ exp. = 1038.4588; obsd = 1038.4594), and (C) MS–MS fragmentation pattern of Glu-C digested His_6_–CaoA core peptide modified by CaoK–CaoY in *E. coli*. For fragment masses, see Table S3.

Previously, the study of lexapeptide biosynthesis demonstrated the ability of LxmKYXD to collectively introduce dehydroamino acids, Lan, and AviMeCys into the precursor peptide LxmA through heterologous co-expression in *Escherichia coli*.^4^*In vivo* investigation of the biosynthesis of the RiPP thioviridamide from *Streptomyces* sp. NRRL S-87 also demonstrated that TvaC_S-87_ and TvaD_S-87_, which are homologues of the APH protein LxmK and the HopA1 homologue LxmY, respectively, are capable of dehydration of Ser/Thr residues in the precursor peptide TvaA_S-87_.^12^ Thioviridamide is a member of the thioamitides that contain thioamide residues as the unifying post-translational modification.^1^ Furthermore, TvaC_S-87_ was also identified as sharing similarity to the kinase domain of the class III lanthipeptide synthetase MicKC,^13^ and mutation of the conserved catalytic residues abolished the production of dehydrated product.^12^ Similar results were reported from *in vitro* reconstitution of the biosynthesis of another thiamitide termed thioholgamide.^14^ In the current study, adopting the nomenclature from the *lxm* BGC,^4^ we first replaced the original numerical order-based designation of genes in the *cao* BGC to correspond to their *lxm* homologs (Figure 1B). To verify the kinase activity of CaoK, we co-expressed CaoK with the N-terminally His_6_-tagged precursor peptide CaoA (His_6_–CaoA) in *E. coli*. The peptide was purified by immobilized metal affinity chromatography (IMAC). Analysis by matrix-assisted laser desorption/ionization time-of-flight mass spectrometry (MALDI-TOF MS) revealed multiple peaks along with the ion of the precursor peptide, with mass shifts corresponding to peptides that had been phosphorylated up to four times (Figure 2A). We then introduced the putative lyase CaoY into the co-expression system resulting in predominantly eight dehydrations of CaoA, consistent with the structure of mature cacaoidin (Figure 2A).^3^ Mass spectral analysis also illustrated the presence of glutathione (GSH) addition, likely due to the large number of dehydroalanines in the modified core peptide, which complicated the *in vivo* analysis of CaoK and CaoY. We therefore focused on reconstituting the activity of CaoK and CaoY *in vitro*.

Initially, N-terminally His_6_-tagged CaoK and CaoY were expressed individually in *E. coli*, but His_6_–CaoK was found mostly in the insoluble portion after cell lysis. Its solubility increased when it was co-expressed with untagged CaoY. His_6_–CaoK and CaoY co-eluted as an enzyme complex during IMAC purification (Figures S1 and S2). Similar results were very recently reported for the dehydratase composed of ThoCD involved in thioholgamide biosynthesis.^14^ Size exclusion chromatography (SEC) purification indicated the formation of a heterodimer composed of His_6_–CaoK and CaoY. The precursor peptide CaoA was then reacted with the purified CaoK–CaoY complex in the presence of adenosine triphosphate (ATP) and MgCl_2_, resulting in 8-fold dehydrated CaoA as the main product (Figure 2A). The instrument on which the MALDI-TOF mass spectra were recorded has a relatively large error at the mass range of these peptides that do not ionize well. Therefore, the dehydrated CaoA was treated with endoproteinase Glu-C to release the core peptide (Figure S3), followed by analysis by high-performance liquid chromatography (HPLC) coupled with electrospray ionization-quadrupole-time-of-flight tandem mass spectrometry (LC-ESI-QTOF-MS/MS). The +2 charge state of 8-fold dehydrated CaoA core peptide was observed (Figure 2B), and the dehydration sites were established by MS/MS fragmentation (Figure 2C and Table S2). The data agree with the final structure of cacaoidin.

### Characterization of CaoY

**Figure 3:**
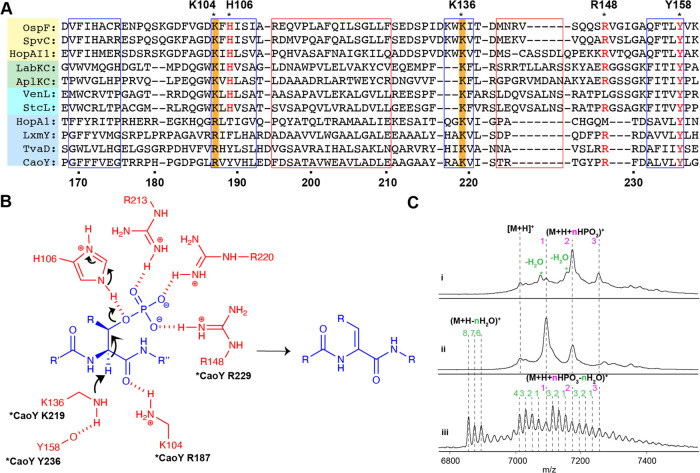
(A) Predicted secondary structure-based sequence alignment analysis of the lyase-like domains of OspF, LanKC/L, and LanY. Predicted α-helix and β-sheet regions are framed in red and blue, respectively. Conserved residues among the three protein families are highlighted in red font. Arg187 and Lys219 studied by mutagenesis are highlighted in orange. Asterisks denote the residues present in the active site in the crystal structure of OspF and in LanKC/L proteins. Residue numbering for SpvC and CaoY is shown at the top and bottom, respectively. (B) Proposed catalytic mechanism for β-elimination of the phosphate of pSer/Thr catalyzed by OspF. The phosphorylated residue is colored in blue, and active site residues are colored in red. Corresponding sites in CaoY (when conserved) are indicated. (C) MALDI-TOF mass spectrum of (i) His_6_–CaoA modified by CaoK–CaoY-R187A in the presence of ATP and MgCl_2_. [M + H]^+^ exp. = 7025, obsd = 7025; (M + H + HPO_3_)^+^ exp. = 7105, obsd = 7104; (M + H + 2HPO_3_)^+^ exp. = 7185, obsd = 7185; (M + H + 3HPO_3_)^+^ exp. = 7265, obsd = 7265; (M + H + 2HPO_3_ – H_2_O)^+^ exp. = 7087, obsd = 7087; (M + H + 3HPO_3_ – H_2_O)^+^ exp. = 7167, obsd = 7167. (ii) His_6_–CaoA modified by CaoK–CaoY-K219A in the presence of ATP and MgCl_2_. [M + H]^+^ exp. = 7025, obsd = 7023; (M + H + HPO_3_)^+^ exp. = 7105, obsd = 7102; (M + H + 2HPO_3_)^+^ exp. = 7185, obsd = 7183. (iii) His_6_–CaoA modified by CaoK–CaoY-R229Q in the presence of ATP and MgCl_2_. (M + H – 8H_2_O)^+^ exp. = 6881, obsd = 6880; (M + H – 7H_2_O)^+^ exp. = 6899, obsd = 6898; (M + H + HPO_3_ – 3H_2_O)^+^ exp. = 7051, obsd = 7048; (M + H + 2HPO_3_ – 3H_2_O)^+^ exp. = 7131, obsd = 7127.

Previous bioinformatics studies demonstrated sequence similarity between CaoY and HopA1 (PF17914),^3,7^ a *Pseudomonas syringae* effector protein that suppresses the effector-triggered immunity response in plants by targeting the positive immune regulator enhanced disease susceptibility1 (EDS1).^15,16^ An *in vivo* kinase-effector interaction screen also identified a number of other kinases as putative HopA1 targets, many of which are shared with other *P. syringae* effectors, such as HopAI1,^17^ implying a similar protein–protein interaction mechanism adopted by these two effectors. HopAI1 suppresses pathogen-associated molecular patterns-induced immunity of the plant host by inactivating the mitogen-activated protein kinase (MAPK) cell signaling pathway.^18^ HopAI1 inactivates MAPKs through elimination of the phosphate group from phosphothreonine (pThr) on their kinase activation loop. This activity resembles that of the OspF family of proteins during infection of mammals (e.g., OspF from *Shigella* and SpvC from *Salmonella*).^19,20^ Sequence alignment of the OspF family proteins with the lyase domain of the class III and IV lanthionine synthetases LanKC and LanL illustrated the high conservation of essential catalytic residues, suggesting that OspF family proteins and LanKC/LanL may have evolved from a common primitive pSer/pThr lyase.^19,21−23^ However, previous sequence alignment of other HopA1-like proteins in RiPP biosynthesis with OspF family proteins and LanKC/LanL did not show significant sequence similarity.^12^ In this study, CaoY and other LanY homologues were aligned with HopA1, HopAI1, OspF family proteins, and LanKC/LanL based on predicted secondary structure.^24^ CaoY and its homologues showed similarity to the lyase catalytic domain of OspF family proteins and LanKC/LanL based on the secondary structure in their C-terminal region (Figure 3A). This predicted structural similarity was also reported recently for ThoD involved in thioholgamide biosynthesis.^14^ Lys136 (Lys219 in CaoY) and Tyr158 (Tyr236 in CaoY) of SpvC that are involved in deprotonation of the α-proton of pSer/pThr residues as revealed by structural studies^20,25^ are highly conserved in all three protein families, whereas Lys104 and His106 are missing in CaoY and its homologues. His106 acts as a catalytic acid to facilitate C_β_-OP cleavage in OspF/SpvC and LanKC/L (Figure 3B). Interestingly, Lys104 that activates the α-proton by stabilizing the oxygen of the enolate formed during phosphate elimination in both OspF/SpvC and LanKC/L is substituted by Arg187 in CaoY (Figure 3A) even though previous Lys-to-Arg mutation at this position in SpvC almost completely abolished its lyase activity.^20^ The residues that form an arginine-rich pocket in SpvC that interact with the phosphate group in the substrate do not align with CaoY in the predicted secondary structure level alignment, except Arg148 (Arg229 in CaoY) (Figure 3A,B), suggesting that CaoY may adopt a different set of residues to stabilize the leaving group.

Site-directed mutagenesis was conducted on CaoY to confirm the similarity between CaoY and OspF/SpvC implied in the predicted secondary structure. We first replaced Lys219 of CaoY with Ala. The enzyme variant was co-expressed and copurified with wild-type His_6_–CaoK, and subsequently, CaoA was supplied to the enzyme complex for *in vitro* dehydration (Figure S4). Instead of full dehydration, only phosphorylated CaoA was observed after 12 h at room temperature (Figure 3C), indicating the loss of lyase activity of CaoY-K219A. This result agrees well with a previous SpvC study in which the SpvC-K136A variant showed no activity.^20^ We then investigated the importance of Arg187. The dehydratase complex formed by CaoY-R187A and CaoK was reacted with CaoA *in vitro*, and phosphate elimination activity was nearly completely abolished (Figure 3C), with mostly phosphorylation observed. To test the role of Arg229 in elimination, CaoA was reacted with CaoY-R229Q-CaoK, and inefficient dehydration was observed (Figure 3C). Collectively, these results demonstrate the catalytic similarity of LanY, the OspF family proteins, and the LanKC/LanL lyase domain.

The heterodimer dehydratase complex formed by CaoK and CaoY also hints at their potential functional similarity to LanKC and LanL (Figures S2 and S4). Instead of a bifunctional enzyme, the kinase and lyase in class V interact to generate a bifunctional enzyme complex. The co-occurrence of LanK and LanY may be used as a bioinformatic handle for class V lanthipeptide discovery. When CaoY was used as query protein for the construction of a genome neighborhood network (GNN),^26,27^ with 10 neighboring genes upstream and downstream collected, high co-occurrence of CaoY homologues (PF17914) and APH family proteins (PF01636) was observed in diverse genome contexts (Figure S5). Similar findings were also reported in the studies on the *spr* BGC.^5^

### Bioinformatic Study of Class V Lanthipeptide Sequence

We then investigated how the heterodimer dehydratase complex might interact with the substrate. Precursor peptides of lanthipeptides typically contain an N-terminal leader peptide (LP) that is critical for the modifying enzymes to recognize the substrate and a C-terminal core peptide, where the modifications take place.^2^ Although the catalytic domains of CaoK and CaoY show sequence similarity to LanKC/LanL, the substrate binding domain of the class IV lanthionine synthetase SgbL^28^ is absent in CaoK. CaoA also lacks the conserved sequence found in class III and class IV lanthipeptide LPs that is believed to be involved in enzyme–substrate recognition.^23^ Therefore, the mechanism by which LanK–LanY recognize their precursor peptide remains poorly understood.

To target the substrate residues that are potentially involved in leader peptide recognition by the class V dehydratase complex, we generated a multiple sequence alignment (MSA) of putative class V LanA sequences using the CaoY GNN described above.^26,27^ From the BGCs with co-occurring LanK and LanY, ORFs shorter than 110 residues^8^ containing Cys and Ser/Thr in their C-terminal region were collected and aligned. The MSA displayed two types of peptides that show remarkable similarity of overall sequence within each group (type-A and type-B) (Figure S6), with a more heterogeneous group that was poorly aligned with the other sequences that we termed type-C.

Interestingly, while type-B potential LanA ORFs are all from cyanobacteria, type-A LanA ORFs were all found in genomes of actinobacteria and especially *Streptomyces*, which encode a large number of lanthipeptides.^29^ All three characterized class V lanthipeptides thus far, including cacaoidin, fall into type-A.^3−5^ In type-A peptides, Tyr–17 in the CaoA leader peptide is fully conserved (Figure S6), implying potential importance in substrate recognition (RiPP leader peptides have negative residue numbers counting back from the core peptide, whereas the core peptide has positive numbers, as shown in Figure 1A). Ala–3 and Pro–2 are also highly conserved through the entire group. In contrast, CaoA Met–24, Phe–20, and Glu–1 are replaced by Leu, Tyr, and Ala in most peptides. The negatively charged Glu in position −12 also shows high conservation with Asp substitution in some sequences. C-terminal to this Glu/Asp residue is usually a hydrophobic residue (Leu, Val, Phe, Ile).

### AlphaFold Model for Substrate Recognition by the Dehydratase Complex

**Figure 4:**
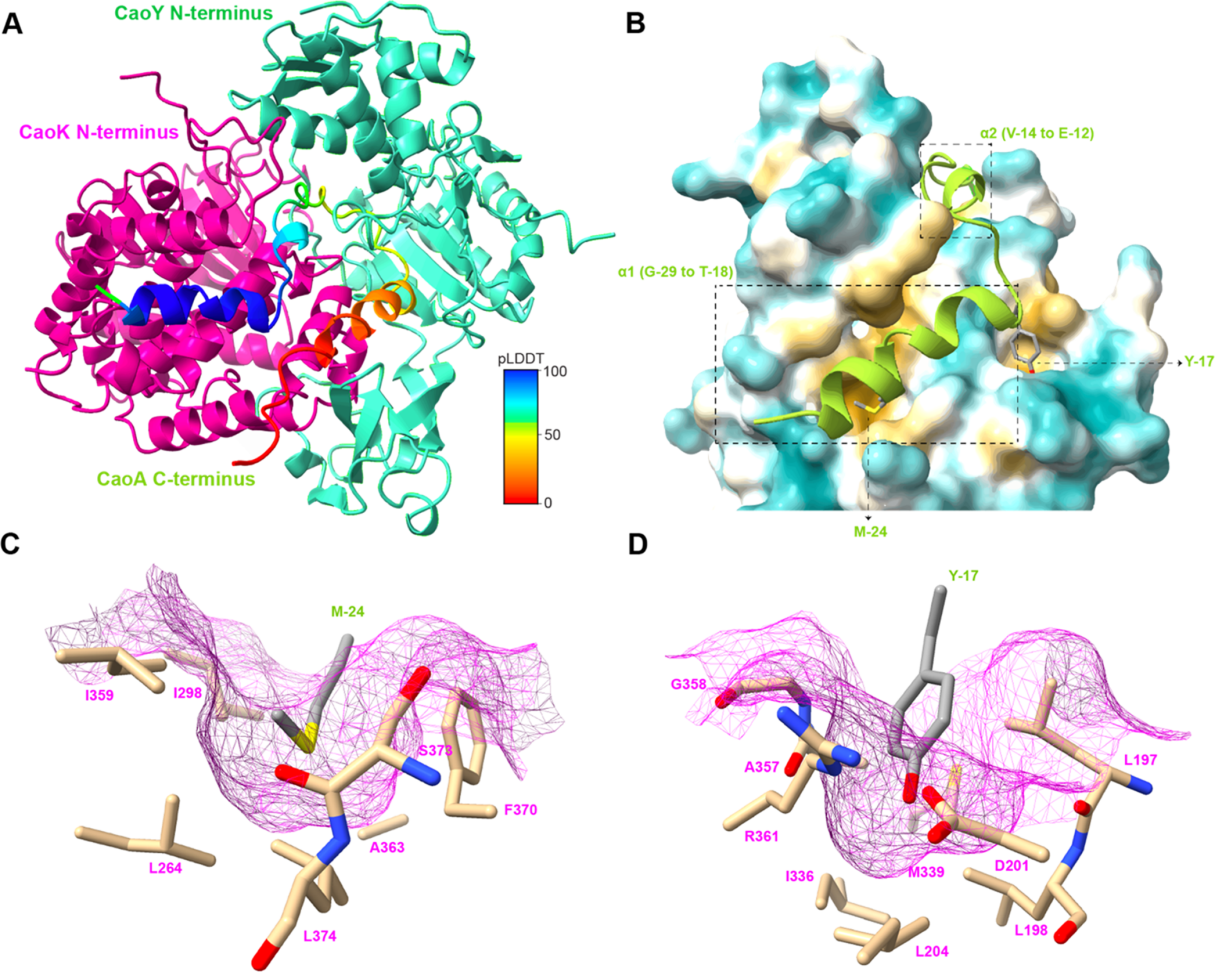
(A) Overall structure of the rank_1 model from AlphaFold-Multimer prediction of the CaoAKY complex. CaoA is colored by pLDDT values (Table S5). CaoK and CaoY are colored in magenta and cyan, respectively. (B) Potential interaction region between CaoA LP and CaoK hydrophobic groove in the predicted structure, with hydrophobic surface in yellow and hydrophilic surface in blue. CaoA is colored in green. The two predicted α-helices, Met–24 and Tyr–17 in the CaoA LP, are depicted. (C, D) Formation of hydrophobic pockets of CaoK that are predicted to capture CaoA LP residues Met–24 (C) and Tyr–17 (D), with CaoK surface in magenta and LP residues of CaoA in gray.

To further investigate the enzyme–substrate interaction, AlphaFold-Multimer was used to generate a predicted structure of the dehydratase complex CaoK–CaoY with the substrate CaoA.^9−11^ We recently showed for the enzyme TglHI, which performs post-translational modifications on the peptide TglA, that the predicted substrate engagement mechanism was close to that reported crystallographically for a closely related enzyme after the AlphaFold model was made.^30,31^ The resulting predicted model of CaoK–CaoY had an average predicted local distance difference test (pLDDT) ranging from 84 to 85.6,^32^ suggesting high accuracy of the overall prediction (Figure S7A,B and Table S5). In the predicted CaoY structure, the previously discussed lyase active site residues Arg187 and Lys219 are in close proximity, lending support to the predicted structure (Figure S8C). Three additional positively charged residues His57, Arg217, and Arg229 are also located around the active site, indicating their potential involvement in catalyzing phosphate elimination (Figure S8D). Alignment of the top-five models demonstrates a consistent prediction of how CaoK, CaoY, and the LP of CaoA interact (Figure S7C,D). In the model, the LP makes contact with the kinase CaoK but not the lyase CaoY. The C-terminus of CaoA (Ile–8 to Cys23) is in different orientations in the five models, with pLDDT values lower than 50 of each residue (Figures 4A, S7, and Table S5), consistent with the movement of the core peptide between the kinase and lyase domains. In the highest-ranking model (model rank_1), the CaoA LP forms two α-helices: One from Gly–29 to Thr–18, and the other from Val–14 to Glu–12 (Figure 4A,B). Both helices have hydrophilic residues exposed to solvent and hydrophobic side chains oriented toward CaoK (Figure 4B).

The hydrophobic face of the first α-helix (Gly–29 to Thr–18) interacts in the model with a hydrophobic groove shaped by helices α13−α16 of CaoK, with Met–24 inserted into a hydrophobic pocket of CaoK made up of Leu264, Ile298, Ile359, Ala363, Phe370, Ser373, and Leu374 (Figures 4C and S8A). The aromatic ring of the side chain of CaoA Phe–20 is predicted to interact with a pocket composed of Val287, Val290, and Leu291 on CaoK (Figure S9A). The side chain of Tyr–17 in the LP of CaoA occupies another pocket formed by Leu197, Leu198, Asp201, Leu204, Ile336, Met339, Leu349, Ala357, and Gly358 of CaoK (Figure 4D). The hydroxyl group of Tyr–17 is predicted to engage in a hydrogen bond with Asp201 of CaoK. Tyr–17 is situated at the end of the helix, and its interaction with the pocket on CaoK pivots the LP such that it makes a 90° turn back into the CaoK hydrophobic groove. An electrostatic interaction between Glu–12, the last residue of the second helix of CaoA that is highly conserved (Figure S6), and Arg354 of CaoK may be functionally important (Figure S9B). After Leu–11, the predictions have low confidence values, and the model is not able to provide reliable information on core peptide binding (Figure S9C).

The interactions between CaoK and CaoY in the complex are mostly mediated by two anti-parallel β-sheets with winged helix motifs (Pro102 to Leu141) on CaoY that interact with three α-helices of CaoK (Leu173 to Gln199) (Figure S8B).

### Site-Directed Mutagenesis of CaoA and CaoY

The functional importance of the residues in the LP of CaoA was probed by site-directed mutagenesis to test the AlphaFold-Multimer prediction (Table 1). Given the highly insoluble nature of the precursor peptide that precluded direct binding studies and kinetic investigations, we chose modification in *E. coli* as a proxy for substrate recognition. His_6_–CaoA variants were co-expressed with wild-type CaoK and CaoY, and the resultant peptides were analyzed by MALDI-TOF MS after purification (Table 1 and Figure S10). In the first helix region of CaoA (Gly–29 to Thr–18), replacement of Met–24 with Gln, a residue with a polar uncharged side chain of comparable size as that of Met, resulted in incomplete dehydration with a range of products observed that had undergone different extents of dehydration and a single phosphorylation (Figure S10). These data suggest that phosphorylation and elimination were affected by the substitution. Replacement of Phe–20 with Gln also resulted in incomplete dehydration, again, with the observation of partially dehydrated and singly phosphorylated peptides. No dehydration was observed when the fully conserved Tyr–17 was substituted by Ala or Gln, with a small amount of phosphorylated peptide observed for the Y–17A variant (Figure S10). Thus, it appears that the engagement of the aromatic ring of Tyr–17 with the CaoK pocket in the model plays an essential role in substrate recognition. In the second helix (Val–14 to Glu–12), replacement of Glu–12 by a positively charged Lys resulted in CaoA that was partially dehydrated, and a similar result was obtained for the variant CaoA-L–11N (Figure S10), which supports the functional importance of the electrostatic interaction between Glu–12 of CaoA and Arg354 of CaoK and the interaction between substrate and kinase mediated by the hydrophobic residue that always follows Glu–12.

**Table 1:**
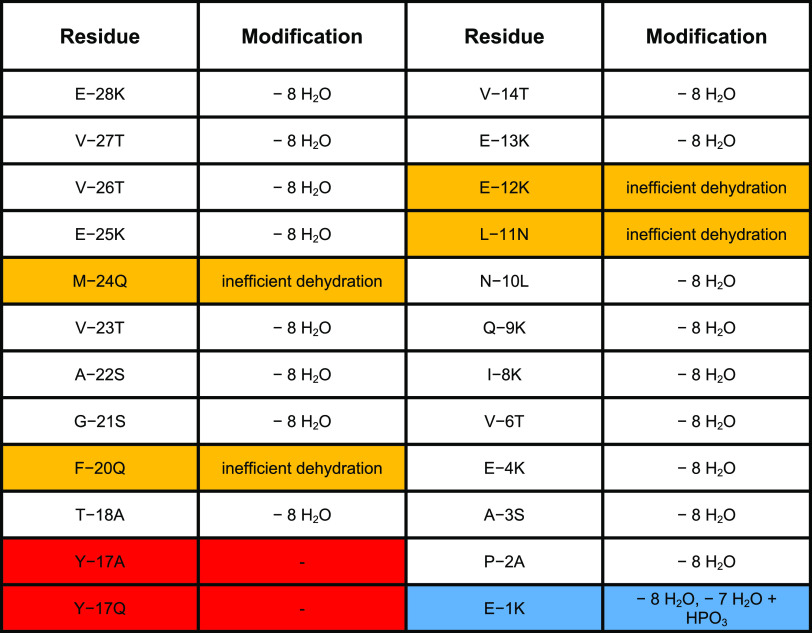
Modification of Variants of CaoA after Co-Expression with CaoK and CaoY^a^

a Mutation of residues that decrease dehydration efficiency are highlighted in orange, that abolish dehydration in red, and that hinder N-terminal phosphate elimination in blue.

Replacement of residues that showed low confidence values in the predicted model or that are not conserved generally had no influence on enzymatic modification, providing 7–8 dehydrations even for nonconservative substitutions (Table 1 and Figure S10; sometimes the dehydrated residues react in the cell with glutathione as previously observed).^33^ These observations are consistent with the model, as these residues mostly face away from the interaction interface between CaoA and CaoK. Thus, Met–24, Phe–20, Tyr–17, Glu/Asp–12, and Leu–11 appear to be the critical residues consistent with the sequence alignment and/or the AlphaFold model. For the LP variants at these positions, the phenotype was either inactive (Tyr–17) or production of a range of dehydration states with one phosphorylation.

One mutation that provided a different phenotype was substitution of Glu–1 with Lys, which resulted in fully dehydrated CaoA along with 7-fold dehydrated phosphorylated CaoA. This observation suggests that the final phosphate elimination is impeded by this substitution. We treated the product with endoproteinase Glu-C to release the core peptide for LC-MS analysis (Figures S11 and S12). Although MS/MS analysis did not allow determination of the specific phosphorylated site, the fragmentation pattern clearly demonstrated that the site was on the N-terminus of the CaoA core peptide (Ser1 to Thr6). We postulate that the phosphorylated residue in the CaoA-E–1K variant was Ser1 and that the replacement of Glu–1 with Lys in CaoA at the junction between the LP an CP resulted in a repulsive interaction between Lys–1 and the two positively charged amino acids in the lyase active site (Arg187 and Lys219). This putative deleterious interaction only seems to hinder the elimination activity of a phosphorylated residue at the N-terminus of the CP as the main product was successfully dehydrated seven times (Figures S10 and S11). This hypothesis also implies C- to N-terminal directionality of the dehydration events catalyzed by CaoK and CaoY.

The AlphaFold model was also leveraged to identify additional CaoY active site residues. As noted above, His57 and Arg229 are close to each other, while Arg217 sits on the opposite side with its side chain oriented away from the proposed active site (Figure S8D). Mutagenesis revealed that whereas CaoY-R217Q retained full activity, mutation of His57 to Asn only resulted in partial dehydration (Figure S13). This compromised function suggests that His57 might work along with Arg229 in stabilizing the phosphate group during elimination. Mutagenesis of Asp201 of CaoK (D201K; D201M; D201W; D201F) to investigate its importance in engaging the phenolic group of Tyr–17 did not lead to the isolation of a CaoKY complex with only insoluble CaoY observed. Similarly, attempts to block the binding site for Met–24 by engineering a salt bridge (I359E/F370K and I359E/L374K) were unsuccessful and also did not result in the isolation of a CaoKY complex.

## Discussion

In this study, we provide detailed insight into dehydration of class V lanthipeptides through reconstitution of the activities of CaoK and CaoY from the cacaoidin biosynthetic pathway. The co-expression and *in vitro* experiments clearly demonstrated kinase and lyase activity of CaoK and CaoY, respectively. A heterodimeric dehydratase complex is formed by CaoK and CaoY, which dehydrated CaoA eight times at the positions consistent with mature cacaoidin. The cooperative behavior of the kinase and lyase illustrates the functional similarity between class V dehydratase and class III-IV lanthionine synthetases LanKC/LanL. Indeed, an evolutionary connection of LanY to the OspF family proteins and the lyase domains of LanKC/LanL was clearly revealed through mutagenesis studies based on a predicted secondary structure alignment. Like OspF/LanL enzymes, LanY likely utilizes a Lys for deprotonation of the α-proton of the pSer/pThr residues, but distinct from the other two families, it uses an Arg instead of Lys for activation of the carbonyl group of the pSer/pThr. The residues that facilitate phosphate elimination through stabilization of the leaving group in OspF/LanL are mostly absent in LanY with only Arg229 conserved. It is likely that at least one additional residue (His57) assists Arg229 in leaving group stabilization.

An AlphaFold-Multimer prediction model of the substrate recognition mechanism adopted by class V lanthipeptide dehydratases was supported by mutagenesis experiments. MSA of putative class V lanthipeptide precursor peptides suggests that this mechanism of substrate recognition is conserved among type-A class V lanthipeptides. Cacaoidin and lexapeptide, two currently characterized class V lanthipeptides, both display strong antimicrobial activity,^3,4,6^ indicating the potential of class V lanthipeptides as a resource of novel antibiotics. Our study on the dehydratase of cacaoidin biosynthesis paves the way for further investigation of class V lanthipeptide biosynthesis as well as the biosynthesis of thioamitides that use a similar complex for dehydration.^12,14^

## Supporting Information Available

Detailed procedures for cloning, protein expression, purification, and activity assays (Figures S1–S13 and Tables S1–S5) (PDF)

## Accession Codes

NCBI Protein accessions: CaoK, QKI29069; CaoY, QKI29067; and CaoA, QKI29070.

## References

[ref1] Montalbán-López M.; Scott T. A.; Ramesh S.; Rahman I. R.; van Heel A. J.; Viel J. H.; Bandarian V.; Dittmann E.; Genilloud O.; Goto Y.; et al. New developments in RiPP discovery, enzymology and engineering. Nat. Prod. Rep. 2021, 38, 130–239. 10.1039/D0NP00027B. 32935693 PMC7864896

[ref2] Repka L. M.; Chekan J. R.; Nair S. K.; van der Donk W. A. Mechanistic understanding of lanthipeptide biosynthetic enzymes. Chem. Rev. 2017, 117, 5457–5520. 10.1021/acs.chemrev.6b00591. 28135077 PMC5408752

[ref3] Ortiz-López F. J.; Carretero-Molina D.; Sánchez-Hidalgo M.; Martín J.; González I.; Román-Hurtado F.; de la Cruz M.; García-Fernández S.; Reyes F.; Deisinger J. P.; et al. Cacaoidin, first member of the new lanthidin RiPP family. Angew. Chem., Int. Ed. 2020, 59, 12654–12658. 10.1002/anie.202005187. 32407589

[ref4] Xu M.; Zhang F.; Cheng Z.; Bashiri G.; Wang J.; Hong J.; Wang Y.; Xu L.; Chen X.; Huang S. X.; et al. Functional genome mining reveals a class V lanthipeptide containing a D-amino acid introduced by an F420H2-dependent reductase. Angew. Chem., Int. Ed. 2020, 59, 18029–18035. 10.1002/anie.202008035. 32648341

[ref5] Kloosterman A. M.; Cimermancic P.; Elsayed S. S.; Du C.; Hadjithomas M.; Donia M. S.; Fischbach M. A.; van Wezel G. P.; Medema M. H. Expansion of RiPP biosynthetic space through integration of pan-genomics and machine learning uncovers a novel class of lanthipeptides. PLoS Biol. 2020, 18, e3001026 10.1371/journal.pbio.3001026. 33351797 PMC7794033

[ref6] Román-Hurtado F.; Sánchez-Hidalgo M.; Martín J.; Ortiz-López F. J.; Genilloud O. Biosynthesis and heterologous expression of cacaoidin, the first member of the lanthidin family of RiPPs. Antibiotics 2021, 10, 403 10.3390/antibiotics10040403. 33917820 PMC8068269

[ref7] Finn R. D.; Coggill P.; Eberhardt R. Y.; Eddy S. R.; Mistry J.; Mitchell A. L.; Potter S. C.; Punta M.; Qureshi M.; Sangrador-Vegas A.; Salazar G. A.; Tate J.; Bateman A. The Pfam protein families database: towards a more sustainable future. Nucleic Acids Res. 2016, 44, D279–D285. 10.1093/nar/gkv1344. 26673716 PMC4702930

[ref8] Arnison P. G.; Bibb M. J.; Bierbaum G.; Bowers A. A.; Bugni T. S.; Bulaj G.; Camarero J. A.; Campopiano D. J.; Challis G. L.; Clardy J.; et al. Ribosomally synthesized and post-translationally modified peptide natural products: overview and recommendations for a universal nomenclature. Nat. Prod. Rep. 2013, 30, 108–160. 10.1039/C2NP20085F. 23165928 PMC3954855

[ref9] Jumper J.; Evans R.; Pritzel A.; Green T.; Figurnov M.; Ronneberger O.; Tunyasuvunakool K.; Bates R.; Žídek A.; Potapenko A.; et al. Highly accurate protein structure prediction with AlphaFold. Nature 2021, 596, 583–589. 10.1038/s41586-021-03819-2. 34265844 PMC8371605

[ref10] Mirdita M.; Schütze K.; Moriwaki Y.; Heo L.; Ovchinnikov S.; Steinegger M. ColabFold - Making protein folding accessible to all. Nat. Methods 2022, 19, 679–682. 10.1038/s41592-022-01488-1. 35637307 PMC9184281

[ref11] Evans R.; O’Neill M.; Pritzel A.; Antropova N.; Senior A.; Green T.; Žídek A.; Bates R.; Blackwell S.; Yim J. Protein complex prediction with AlphaFold-Multimer bioRxiv 2021, 10.1101/2021.10.04.463034.

[ref12] Qiu Y.; Liu J.; Li Y.; Xue Y.; Liu W. Formation of an aminovinyl-cysteine residue in thioviridamides occurs through a path independent of known lanthionine synthetase activity. Cell Chem. Biol. 2021, 28, 675–685. 10.1016/j.chembiol.2020.12.016. 33476565

[ref13] Wiebach V.; Mainz A.; Siegert M. J.; Jungmann N. A.; Lesquame G.; Tirat S.; Dreux-Zigha A.; Aszodi J.; Le Beller D.; Süssmuth R. D. The anti-staphylococcal lipolanthines are ribosomally synthesized lipopeptides. Nat. Chem. Biol. 2018, 14, 652–654. 10.1038/s41589-018-0068-6. 29915235

[ref14] Sikandar A.; Lopatniuk M.; Luzhetskyy A.; Müller R.; Koehnke J. Total in vitro biosynthesis of the thioamitide thioholgamide and investigation of the pathway. J. Am. Chem. Soc. 2022, 144, 5136–5144. 10.1021/jacs.2c00402. 35263083

[ref15] Dahale S. K.; Ghosh D.; Ingole K. D.; Chugani A.; Kim S. H.; Bhattacharjee S. HopA1 effector from *Pseudomonas syringae* pv syringae strain 61 affects nmd processes and elicits effector-triggered immunity. Int. J. Mol. Sci. 2021, 22, 7440 10.3390/ijms22147440. 34299060 PMC8306789

[ref16] Bhattacharjee S.; Halane M. K.; Kim S. H.; Gassmann W. Pathogen effectors target Arabidopsis EDS1 and alter its interactions with immune regulators. Science 2011, 334, 1405–1408. 10.1126/science.1211592. 22158819

[ref17] Brauer E. K.; Popescu G. V.; Singh D. K.; Calvino M.; Gupta K.; Gupta B.; Chakravarthy S.; Popescu S. C. Integrative network-centric approach reveals signaling pathways associated with plant resistance and susceptibility to *Pseudomonas syringae*. PLoS Biol. 2018, 16, e2005956 10.1371/journal.pbio.2005956. 30540739 PMC6322785

[ref18] Zhang J.; Shao F.; Li Y.; Cui H.; Chen L.; Li H.; Zou Y.; Long C.; Lan L.; Chai J.; et al. A *Pseudomonas syringae* effector inactivates MAPKs to suppress PAMP-induced immunity in plants. Cell Host Microbe 2007, 1, 175–185. 10.1016/j.chom.2007.03.006. 18005697

[ref19] Li H.; Xu H.; Zhou Y.; Zhang J.; Long C.; Li S.; Chen S.; Zhou J. M.; Shao F. The phosphothreonine lyase activity of a bacterial type III effector family. Science 2007, 315, 1000–1003. 10.1126/science.1138960. 17303758

[ref20] Zhu Y.; Li H.; Long C.; Hu L.; Xu H.; Liu L.; Chen S.; Wang D. C.; Shao F. Structural insights into the enzymatic mechanism of the pathogenic MAPK phosphothreonine lyase. Mol. Cell 2007, 28, 899–913. 10.1016/j.molcel.2007.11.011. 18060821

[ref21] Goto Y.; Li B.; Claesen J.; Shi Y.; Bibb M. J.; van der Donk W. A. Discovery of unique lanthionine synthetases reveals new mechanistic and evolutionary insights. PLoS Biol. 2010, 8, e1000339 10.1371/journal.pbio.1000339. 20351769 PMC2843593

[ref22] Goto Y.; Ökesli A.; van der Donk W. A. Mechanistic studies of Ser/Thr dehydration catalyzed by a member of the LanL lanthionine synthetase family. Biochemistry 2011, 50, 891–898. 10.1021/bi101750r. 21229987 PMC3031989

[ref23] Hegemann J. D.; Süssmuth R. D. Matters of class: coming of age of class III and IV lanthipeptides. RSC Chem. Biol. 2020, 1, 110–127. 10.1039/D0CB00073F. 34458752 PMC8341899

[ref24] Söding J.; Biegert A.; Lupas A. N. The HHpred interactive server for protein homology detection and structure prediction. Nucleic Acids Res. 2005, 33, W244–W248. 10.1093/nar/gki408. 15980461 PMC1160169

[ref25] Chen L.; Wang H.; Zhang J.; Gu L.; Huang N.; Zhou J. M.; Chai J. Structural basis for the catalytic mechanism of phosphothreonine lyase. Nat. Struct. Mol. Biol. 2008, 15, 101–102. 10.1038/nsmb1329. 18084305

[ref26] Zhao S.; Sakai A.; Zhang X.; Vetting M. W.; Kumar R.; Hillerich B.; San Francisco B.; Solbiati J.; Steves A.; Brown S.; et al. Prediction and characterization of enzymatic activities guided by sequence similarity and genome neighborhood networks. eLife 2014, 3, e03275 10.7554/eLife.03275. PMC4113996 24980702

[ref27] Gerlt J. A.; Bouvier J. T.; Davidson D. B.; Imker H. J.; Sadkhin B.; Slater D. R.; Whalen K. L. Enzyme Function Initiative-Enzyme Similarity Tool (EFI-EST): A web tool for generating protein sequence similarity networks. Biochim. Biophys. Acta, Proteins Proteomics 2015, 1854, 1019–1037. 10.1016/j.bbapap.2015.04.015. PMC4457552 25900361

[ref28] Hegemann J. D.; Shi L.; Gross M. L.; van der Donk W. A. Mechanistic studies of the kinase domains of class IV lanthipeptide synthetases. ACS Chem. Biol. 2019, 14, 1583–1592. 10.1021/acschembio.9b00323. 31243957 PMC6642009

[ref29] Walker M. C.; Eslami S. M.; Hetrick K. J.; Ackenhusen S. E.; Mitchell D. A.; van der Donk W. A. Precursor peptide-targeted mining of more than one hundred thousand genomes expands the lanthipeptide natural product family. BMC Genomics 2020, 21, 387 10.1186/s12864-020-06785-7. 32493223 PMC7268733

[ref30] McLaughlin M. I.; Yu Y.; van der Donk W. A. Substrate recognition by the peptidyl-(S)-2-mercaptoglycine synthase TglHI during 3-thiaglutamate biosynthesis. ACS Chem. Biol. 2022, 17, 930–940. 10.1021/acschembio.2c00087. 35362960 PMC9016710

[ref31] Dou C.; Long Z.; Li S.; Zhou D.; Jin Y.; Zhang L.; Zhang X.; Zheng Y.; Li L.; Zhu X.; et al. Crystal structure and catalytic mechanism of the MbnBC holoenzyme required for methanobactin biosynthesis. Cell Res. 2022, 32, 302–314. 10.1038/s41422-022-00620-2. 35110668 PMC8888699

[ref32] Mariani V.; Biasini M.; Barbato A.; Schwede T. lDDT: a local superposition-free score for comparing protein structures and models using distance difference tests. Bioinformatics 2013, 29, 2722–2728. 10.1093/bioinformatics/btt473. 23986568 PMC3799472

[ref33] Bothwell I. R.; Caetano T.; Sarksian R.; Mendo S.; van der Donk W. A. Structural analysis of class I lanthipeptides from *Pedobacter lusitanus* NL19 reveals an unusual ring pattern. ACS Chem. Biol. 2021, 16, 1019–1029. 10.1021/acschembio.1c00106. 34085816 PMC9845027

