# Supplementary material for: Mechanistic Studies on Dehydration in Class V Lanthipeptides
Source: ACS Chem Biol. 2022 Aug 31;17(9):2519–27. doi: 10.1021/acschembio.2c00458 (PMC9486802; doi:10.1021/acschembio.2c00458)
Supplement: Supplementary file 1 — cb2c00458_si_001.pdf [file cb2c00458_si_001.pdf]

Supporting Information

Mechanistic studies on dehydration in class V lanthipeptides

Haoqian Liang,[a] Isaiah J. Lopez,[a] Marina Sánchez-Hidalgo,[c] Olga Genilloud,[c] and Wilfred A. van der Donk[a,b,*]

[a] Department of Biochemistry, University of Illinois at Urbana–Champaign, 600 S. Mathews Ave, Urbana, IL 61801, USA
[b] Department of Chemistry and Howard Hughes Medical Institute, University of Illinois at Urbana–Champaign, 600 S. Mathews Ave, Urbana, IL 61801, USA
[c] Fundación MEDINA, Centro de Excelencia en Investigación de Medicamentos Innovadores en Andalucía, Avenida del Conocimiento, 34 Parque Tecnológico de Ciencias de la Salud, 18016 Armilla, Granada, Spain

[*] To whom correspondence should be addressed: vddonk@illinois.edu

Materials and Methods

Primers and gBlocks were ordered through TWIST Bioscience and Integrated DNA Technologies. Endoproteinase Glu-C and Q5 HF DNA Polymerase were purchased from New England Biolabs. ATP disodium trihydrate was purchased from Sigma-Aldrich. Sinapic acid and super DHB (a mixture of 2,5-dihydroxybenzoic acid and 2-hydroxy-5-methoxybenzoic acid) for use as matrices in MALDI-TOF MS were purchased from Sigma-Aldrich. The 4%-20% precast polyacrylamide gels used for Sodium Dodecyl-Sulfate PolyacrylAmide Gel Electrophoresis (SDS-PAGE) were purchased from Bio-Rad. Prestained Rec Protein ladder was purchased from Fisher Scientific. DNA miniprep, PCR purification, and gel extraction kits were purchased from Qiagen. The Amicon centrifugal filters used for protein and peptide concentration were manufactured by EMD Millipore. PCR was performed using a Bio-Rad C1000 thermocycler. HisTrap resin/columns for protein and peptide purification were

purchased from GE Healthcare. Protein purification was conducted on an Amersham Biosciences ÄKTApurifier fast protein liquid chromatography (FPLC) system purchased from GE Healthcare. Size exclusion chromatography (SEC) purification was conducted using SuperdexTM 75 10/300 GL. High-resolution mass spectrometry (HRMS) was performed on an Agilent 6545 LC-ESI-QTOF with Kinetex® 2.6 µm C8 100 Å LC Column 150 x 2.1 mm. HRMS data were analyzed using Agilent MassHunter. MALDI-TOF MS analysis was carried out in the mass spectrometry facility at the University of Illinois at Urbana–Champaign (UIUC) using a Bruker Daltonics UltrafleXtreme MALDI TOF mass spectrometer. MALDI-TOF MS data were calibrated using Protein Calibration Standard I purchased from Bruker and processed using the software FlexAnalysis. AlphaFold-Multimer predicted structure model was performed with Google Colab and analyzed either with Pymol or Chimera X.[1-6] Generation of the sequence similarity network (SSN) and genome neighborhood network (GNN) were carried out by using the EFI web tools and data were analyzed using Cytoscape.[7, 8]

Experimental Procedures

Cloning

All primers and gBlocks used for cloning and sequencing are listed in Table S1. His_6-CaoA and variants were cloned into pACYCDuet-1 multi cloning site II (MCSII) by Gibson assembly. His_6-CaoA was cloned into pRSFDuet-1 MCSII and CaoY or CaoK was cloned into pRSFDuet-1 MCSI for CaoK/Y-CaoA co-expression. CaoY was cloned into pRSFDuet-1 MCSI and CaoK (tagged or untagged) in MCSII. *E. coli* DH10β was used as the host for plasmid propagation, and DNA sequencing to confirm successful

cloning was completed by ACGT, Inc. For MCSI, sequencing was completed using an ACYCDuetUP1 and DuetDOWN1 primer supplied by ACGT, Inc. For MCSII, sequencing was completed with DuetUP2 and T7 term.

Overexpression and purification

When His$_6$-CaoA was expressed alone, *E. coli* NovaBlue T1^R Singles™ competent cells transformed with pACYCDuet-1-His$_6$-CaoA (MCSII) were inoculated in 10 mL of LB with 35 μg/mL chloramphenicol. This culture was used to inoculate 1 L of LB media containing 35 μg/mL chloramphenicol to an initial O.D. at 600 nm of 0.03. The cultures were grown at 37 ^oC with shaking at 220 RPM to an O.D. at 600 nm of 0.6 and overexpression was induced by addition of 1 M IPTG to 1 mM final concentration. After 18 h continued shaking at 18 ^oC, the cultures were centrifuged for 15 min at 4000 × g at 4 ^oC, and the cell pellet was resuspended in LanA buffer B1(6 M guanidine HCl, 20 mM NaH_2PO_4, 500 mM NaCl, 0.5 mM imidazole, pH 7.5) and lysed by sonication (75% amplitude, 5 min, 2.0 s pulse on, 5.0 s pulse off), then centrifuged at 49000 × g for 20 min at 4 ^oC. The supernatant was loaded on 2 mL of packed Ni-NTA His•Bind® Resin in LanA buffer B1. The resin was washed with LanA buffer B2 (4 M guanidine HCl, 20 mM NaH_2PO_4, 300 mM NaCl, 30 mM imidazole, pH 7.5) and peptides were eluted in LanA buffer EB (4 M guanidine HCl, 20 mM Tris-HCl, 100 mM NaCl, 1 mM imidazole, pH 7.5). The elution fraction was collected and concentrated. The concentration was determined on a NanoDrop based on the A280 nm absorption using the extinction coefficient predicted by Expasy. The sample was ziptipped with an EMD Millipore C18 ziptip, eluting from the ziptip into 5 μL of 1:1 MeCN:H_2O, then mixed with 5 μL of saturated sinapic acid in 1:1 MeCN:H_2O and 1 μL was spotted on a MALDI plate. MALDI-TOF MS

analysis was conducted on a Bruker Ultraflex mass spectrometer in linear mode with a 5-20 kDa range. The sample was then stored at -20 ^oC.

For overexpression and purification of pRSFDuet-1-CaoK (MCSI)-His$_6$-CaoA(MCSII) and pRSFDuet-1-CaoY(MCSI)-His$_6$-CaoA(MCSII), the procedure was similar to that described above but using 50 μg/mL kanamycin as antibiotic instead. When both enzymes in pRSFDuet-1 were co-expressed with substrate in pACYCDuet-1, 50 μg/mL kanamycin and 35 μg/mL chloramphenicol were added into the culture.

For protein expression and purification, *E. coli* NovaBlue T1^R Singles™ competent cells transformed with the corresponding pRSFDuet-1 were inoculated in 10 mL of LB with 50 μg/mL kanamycin. This culture was used to inoculate 1 L of LB media containing 50 μg/mL kanamycin to an initial O.D. at 600 nm of 0.03. The cultures were grown at 37 ^oC with shaking at 220 rpm to an O.D. at 600 nm of 0.6 and overexpression was induced by addition of 1 M IPTG to 1 mM final concentration. After 18 h continued shaking at 18 ^oC, the cultures were centrifuged for 15 min at 4000 × g at 4 ^oC, and the cell pellet was resuspended in LanM start buffer (20 mM Tris-HCl, 1 mM NaCl, pH 7.5) and lysed by sonication (35% amplitude, 5 min, 2.0 s pulse on, 5.0 s pulse off), then centrifuged at 49000 × g for 20 min at 4 ^oC. The supernatant was loaded on a HipTrap column and eluted using an FPLC instrument with LanM final buffer (20 mM Tris-HCl, 1 mM NaCl, 200 mM imidazole, pH 7.5). The elution fractions were collected and concentrated and loaded for SEC purification. The elution fractions corresponding to the molecular mass of the desired protein or protein complex (determined by SDS-PAGE) were collected and concentrated to 0.5 mM. The concentration was determined on a NanoDrop based on the A280 absorption using the extinction coefficient predicted by Expasy. For gel

analysis, 0.5 μg of protein elution buffer was diluted with 4x SDS loading buffer. The sample was boiled at 90 ^oC for 10 min then loaded in gel. The gel was run for 50 min at 160 V in fresh 1x Tris glycine SDS buffer, and the protein bands were visualized by Coomassie staining. The protein sample was then flash-frozen and stored at -80 ^oC.

LC-ESI-QTOF analysis of CaoA and CaoA variants

His_6-CaoA and His_6-CaoA-E−1K isolated from co-expressions with CaoK and CaoY were buffer exchanged into 20 mM HEPES buffer with pH 7.5 using 3 k Amicon filters and concentrated. To 100 μL of 20 mM buffer was added 500 ng endoproteinase Glu-C, 50 μL 2x GluC reaction buffer, and 20 μg peptide. After overnight reaction at 37 ^oC, the sample was ziptipped with Glygen TopTip C-08, and eluting from the TopTip into 100 μL of 2% formic acid in MeCN. Then 5 μL of eluted sample was mixed with 5 μL of saturated sinapic acid in 1:1 MeCN:H_2O and 1 μL was spotted on a MALDI plate. MALDI-TOF MS analysis was conducted on a Bruker Ultraflextreme mass spectrometer in linear mode with a 5-20 kDa range.

The peptide in elution buffer was lyophilized and dissolved in HPLC grade water and injected on a Kinetex® C8 LC column and analyzed in positive detection mode on an Agilent 6545 LC-ESI-QTOF with a drying gas temperature of 320 °C and gas flow of 13 L/min, sheath gas temperature of 350 °C and gas flow of 11 L/min.

***In vitro* dehydration assay**

In 50 μL the assay contained 5 μM His_6-CaoK and CaoY (or CaoY variants), 5 μM His_6-CaoA, 5 mM ATP, and 5 mM $MgCl_2$ in 20 mM HEPES buffer (pH 7.5). After overnight reaction at room temperature, the proteins were denatured by adding 50 μL of 2% trifluoroacetic acid (TFA) and centrifuged for 2 min at 14000 × g at room temperature.

The supernatant was ziptipped using EMD Millipore C18 ziptip, and the peptide was eluted and analyzed by MALDI-TOF MS as described above.

Table S1. List of primers and double stranded DNA fragments

DNA Name	Sequence
LL_CaoA_pACYC_2-F	GTTAAGTATAAGAAGGAGATATACATATGGGCAGCAGCCATCACC
LL_CaoA_pACYC_2-R	CGGCCGATATCCAATTGAGATCTGCTCAGCAGCCCCAGGAAGCG
LL_Cao7_pRSF_1-F	GTTTAACTTTAATAAGGAGATATACCATGGTCATTCTTCACGATGCAG
LL_Cao7_pRSF_1-R	GATTACTTTCTGTTCGACTTAATTATTCACCACGGGCCGC
LL_Cao7_pRSF_2-F	GTATAAGAAGGAGATATACATATGGTCATTCTTCACGATGCAGTTAG
LL_Cao7_pRSF_2-R	CTTTACCAGACTCGAGGGTACCGTTATTCACCACGGGCCGC
LL_Cao9_pRSF_2-F	GTATAAGAAGGAGATATACATATGGTTACCGTCTCCGTCAAG
LL_Cao9_pRSF_2-R	CTTTACCAGACTCGAGGGTACCGTTAATTGAGCAAAGGTTCAAGACCC
LL_Cao7_R187A_F1	CGCCACGGCAGTATGGGAAG
LL_Cao7_R187A_R1	CGGGATGAGGACGACGTGTTC
LL_Cao7_R187A_Insert	GAACACGTCGTCCTCATCCCGGTGATCCCGGGTTAGCTGTATACGTCCATCTTGAAGATTTTGATAGCGCCACGGCAGTATGGGAAG
LL_Cao7_K219A_F1	GGGTTACCCACGTTTTGATGCCCTTGTATTATATTTG
LL_Cao7_K219A_R1	GCTCCTGCTGCTTCCAAGTC
LL_Cao7_K219A_Insert	GACTTGGAAGCAGCAGGAGCGGCATATCGTGCGGCGGTGATTTCAACGCGTACGGGTTACCCACGTTTTGATGCCCTTGTATTATATTTG
LL_LPMUT2_F1	CACGCCCAGTCGCG
LL_E6K_Inser_R1	GACGACCTCTCCCATCATCG
LL_E6K_R1	GTGTCGAAGCCGGCCACCATTTTGACGACCTCTCCCATCATCG
LL_E6K_BB_F1	GTCAAAATGGTGGCCGGCTTCG
LL_LPMUT2_BB_R1	CCCATGAAGACGGTACGC
LL_T13A_Inser_R1	GTCGAAGCCGGCCAC
LL_T13A_R1	CTCCTCGACATCCGCGTACGCGTCGAAGCCGGCCAC

LL_T13A_BB_F1	GACGCGTACGCGGATGTCGAGGAG
LL_Y14A_R1	CAGCTCCTCGACATCCGCCGCGGTGTCGAAGCCGGCCAC
LL_Y14A_BB_F1	ACCGCGGCGGATGTCGAGGAGCTG
LL_E19K_Inser_R1	CTCGACATCCGCGTAGGTG
LL_E19K_R1	CCGACCGCGATCTGGTTCAGTTTCTCGACATCCGCGTAGGTG
LL_E19K_BB_F1	GAGAAACTGAACCAGATCGCGGTC
LL_Q22K_R1	GCGCCTCGCCGACCGCGATTTTGTTCAGCTCCTCGACATCCGCGTAGGTG
LL_Q22K_BB_F1	AACAAAATCGCGGTCGGCG
LL_E27K_Inser_R1	GCCGACCGCGATCTG
LL_E27K_R1	GAGCGGAGGACTCCGGCGCTTTGCCGACCGCGATCTG
LL_E27K_BB_F1	GGCAAAGCGCCGGAGTCCTC
LL_E30K_R1	GTGCACGGAGCGGAGGATTTCGGCGCCTCGCCGACCGCGATCTG
LL_E30K_BB_F1	CCGAAATCCTCCGCTCCGTGC
LL_V17T_R1	GGTTCAGCTCCTCGGTATCCGCGTAGGTGTCGAAG
LL_V17T_BB_F1	GACACCTACGCGGATACCGAGGAGCTGAACCAGATCG
LL_E18K_R1	GATCTGGTTCAGCTCTTTGACATCCGCGTAGGTGTCG
LL_E18K_BB_F1	CCTACGCGGATGTCAAAGAGCTGAACCAGATCGCG
LL_L20N_R1	CGACCGCGATCTGGTTATTCTCCTCGACATCCGCGTAG
LL_L20N_BB_F1	GATGTCGAGGAGAATAACCAGATCGCGGTCGG
LL_N21L_R1	CGCCGACCGCGATCTGCAGCAGCTCCTCGACATCCG
LL_N21L_BB_F1	GATGTCGAGGAGCTGCTGCAGATCGCGGTCGGC
LL_I23K_R1	CCTCGCCGACCGCTTTCTGGTTCAGCTCCTCGAC
LL_I23K_BB_F1	GAGGAGCTGAACCAGAAAGCGGTCGGCGAGG
LL_V25T_R1	CTCCGGCGCCTCGCCGGTCGCGATCTGGTTCAGCTC
LL_V25T_BB_F1	GAACCAGATCGCGACCGGCGAGGCGC
LL_A28S_R1	GCGGAGGACTCCGGGCTCTCGCCGACCGCG
LL_A28S_BB_F1	CGCGGTCGGCGAGAGCCCGGAGTCCTCCGC
LL_P29A_R1	CGGAGCGGAGGACTCAGCCGCCTCGCCGACC
LL_P29A_BB_F1	GGTCGGCGAGGCGGCTGAGTCCTCCGCTCCG

LL_LPMUT3_F1	GATGCTCCACGCCCAG
LL_LPMUT2_BB_R1	CCCATGAAGACGGTACGC
LL_V4T_R1	CACCATCTCGACCGTCTCTCCCATCATCGGATCCTG
LL_V4T_BB_F1	CCGATGATGGGAGAGACGGTCGAGATGGTGGCCG
LL_V5T_R1	GCCGGCCACCATCTCCGTGACCTCTCCCATCATCGGATC
LL_V5T_BB_F1	GATGGGAGAGGTCACGGAGATGGTGGCCGGC
LL_M7Q_R1	GGTGTCGAAGCCGGCCACCTGCTCGACGACCTCTCCCATC
LL_M7Q_BB_F1	GGTCGTCGAGCAGGTGGCCGGCTTCG
LL_V8T_R1	GTAGGTGTCGAAGCCGGCGGTCATCTCGACGACCTCTCCC
LL_V8T_BB_F1	CGTCGAGATGACCGCCGGCTTCGACACC
LL_A9S_R1	GCGTAGGTGTCGAAGCCGCTCACCATCTCGACGACCTCTC
LL_A9S_BB_F1	GTCGTCGAGATGGTGAGCGGCTTCGACACCTACG
LL_G10S_R1	CCGCGTAGGTGTCGAAAGAGGCCACCATCTCGACG
LL_G10S_BB_F1	GAGATGGTGGCCTCTTTCGACACCTACGCGGATG
LL_F11Q_R1	CCGCGTAGGTGTCCTGGCCGGCCACCATCTC
LL_F11Q_BB_F1	GAGATGGTGGCCGGCCAGGACACCTACGCGGATGTC
LL_Y14Q_R1	CTCCTCGACATCCGCCTGGGTGTCGAAGCCGGC
LL_Y14Q_BB_F1	CGGCTTCGACACCCAGGCGGATGTCGAGGAGC
LL_E3K_R1	GCCACCATCTCGACGACTTTTCCCATCATCGGATCCTGG
LL_E3K_BB_F1	CCGATGATGGGAAAAGTCGTCGAGATGGTGGC

Table S2. Calculated ppm error of LC-ESI-QTOF of the fragment ions observed in the tandem mass spectrum of Glu-C digested 8-fold dehydrated His_6-CaoA core peptide. Exp. m/z = expected m/z; Obs. m/z = observed m/z.

Mass fragment	Exp. m/z	Obs. m/z	ppm error
$[M+2H]^{2+}$	1038.4588	1038.4594	-0.58
y_{16}^+	1470.6471	1470.6465	0.41
y_{14}^+	840.3709	840.3711	0.24
y_{12}^+	1068.4567	1068.4569	-0.19
y_{11}^+	928.3982	928.3977	0.54
y_{10}^+	746.2926	746.2926	0.00
y_5^+	523.1969	523.1943	-4.97
b_6^+	493.1864	493.1863	0.20
b_7^+	606.2704	606.2709	-0.82
b_8^+	769.3338	769.3342	-0.52
b_9^+	840.3709	840.3711	-0.24
b_{11}^+	1008.4608	1008.4602	0.59

Table S3. Calculated ppm error of fragment ions observed by LC-ESI-QTOF of Glu-C digested 7-fold dehydrated and 1-fold phosphorylated His_6-CaoA-E−1K core peptide.

Mass fragment	Exp. m/z	Obs. m/z	ppm error
$[M+2H]^{2+}$	1235.5396	1235.5391	-0.40
y_8^+	746.2926	746.2897	-3.89
y_{10}^+	928.3982	928.3976	-0.65
y_{16}^+	1470.6471	1470.6432	-2.65
b_3^+	297.1921	297.1926	1.68
b_{10}^+	1000.4322	1000.4305	-1.70
b_{14}^+	1402.6225	1402.6135	-6.42

Table S4. MALDI-TOF MS data of full length His_6-CaoA variants expressed alone and co-expressed with CaoK-CaoY in *E. coli* in Figure S9.

Variants	Exp. m/z of unmodified variants	Obs. m/z of unmodified variants	Exp. m/z of 8-fold dehydrated variants	Obs. m/z of 8-fold dehydrated variants
E−28K	7024	7028	6880	6883
V−27T	7027	7022	6883	6881
V−26T	7027	7022	6883	6879
E−25K	7024	7017	6880	6873
M−24Q	7022	7022	6878	-
V−23T	7027	7027	6883	6884
A−22S	7041	7036	6897	6893
G−21S	7055	7054	6911	6907
F−20Q	7006	7004	6862	-
T−18A	6995	6997	6851	6857
Y−17A	6933	6937	6789	-
Y−17Q	6990	6985	6846	-
V−14T	7027	7023	6883	6880
E−13K	7024	7021	6880	6880
E−12K	7024	7025	6880	-
L−11N	7026	7027	6882	-
N−10L	7024	7023	6880	6873
Q−9K	7025	7019	6881	6885
I−8K	7040	7040	6896	6891
V−6T	7027	7030	6883	6889
E−4K	7024	7027	6880	6884
A−3S	7041	7043	6897	6895
P−2A	6999	6998	6855	6855
E−1K	7024	7018	6880	-

Table S5. Predicted local distance difference test (pLDDT) values of CaoA residues from five highest-ranking CaoAKY predicted structures models (rank_1 to rank_5).

Residue	rank_1	rank_2	rank_3	rank_4	rank_5	Average
M−30	53.70	56.55	57.36	55.04	57.85	56.10
G−29	87.19	87.19	82.89	83.79	82.54	84.72
E−28	91.66	91.95	89.16	89.70	90.55	90.60
V−27	95.01	95.24	93.86	94.84	94.61	94.71
V−26	95.72	95.86	94.44	95.49	95.28	95.36
E−25	94.87	95.12	93.47	94.18	94.18	94.36
M−24	95.30	95.51	93.77	94.23	94.46	94.65
V23	95.75	95.94	94.50	95.73	95.49	95.48
A−22	95.23	95.56	93.53	95.32	94.99	94.93
G−21	94.23	95.36	92.75	94.59	94.25	94.24
F−20	92.07	93.09	90.76	92.57	92.64	92.23
D−19	92.21	92.44	91.02	92.67	92.84	92.24
T−18	93.75	93.74	91.46	93.79	93.67	93.28
Y−17	92.79	93.47	91.00	93.24	92.99	92.70
A−16	87.91	88.43	86.43	87.89	87.49	87.63
D−15	85.91	85.08	84.02	85.25	86.19	85.29
V−14	83.47	82.14	81.21	82.40	82.83	82.41
E−13	76.65	75.59	74.65	75.89	76.48	75.85
E−12	77.42	76.65	75.12	75.03	77.02	76.25
L−11	78.17	76.60	75.36	76.78	76.64	76.71
N−10	63.87	61.07	65.84	65.94	64.02	64.15
Q−9	58.31	54.84	59.36	57.24	59.32	57.81
I−8	47.90	47.40	52.81	47.98	50.41	49.30
A−7	47.54	46.68	47.66	43.09	51.36	47.27
V−6	44.44	39.85	46.64	37.58	57.77	45.26
G−5	36.73	38.22	38.25	35.74	39.95	37.78
E−4	36.15	36.57	37.70	36.63	37.56	36.92
A−3	42.05	37.23	40.09	36.11	41.41	39.38
P−2	40.71	32.72	37.21	34.37	34.52	35.91
E−1	41.38	31.08	39.42	33.21	33.15	35.65
S1	42.82	30.81	41.97	35.38	35.33	37.26
S2	39.23	29.31	39.82	30.66	32.83	34.37
A3	34.24	33.10	35.70	30.08	29.17	32.46
P4	32.50	31.95	36.27	29.63	29.25	31.92

C5	31.72	34.08	34.55	27.38	28.41	31.23
T6	28.67	32.90	31.22	24.53	24.69	28.40
I7	27.79	32.43	27.16	23.57	21.41	26.47
Y8	24.54	36.18	22.94	18.91	18.78	24.27
A9	24.32	32.85	22.48	20.48	20.23	24.07
S10	23.89	31.90	22.34	20.52	20.41	23.81
V11	23.13	31.91	20.50	18.81	19.72	22.81
S12	20.51	30.92	19.77	17.51	17.99	21.34
A13	20.64	31.32	19.86	18.63	18.60	21.81
S14	20.25	29.99	20.04	17.76	19.73	21.55
I15	19.16	28.70	18.59	16.50	17.66	20.12
S16	18.16	28.76	17.31	16.75	16.77	19.55
A17	18.50	28.14	17.80	18.01	17.49	19.99
T18	18.66	28.80	18.44	18.68	17.78	20.47
A19	17.45	27.01	17.31	17.59	18.51	19.57
S20	16.58	24.38	16.31	17.00	17.87	18.43
W21	16.00	20.17	16.34	17.19	18.20	17.58
G22	15.23	19.11	18.53	16.75	18.50	17.62
C23	14.45	19.87	17.44	16.76	17.53	17.21

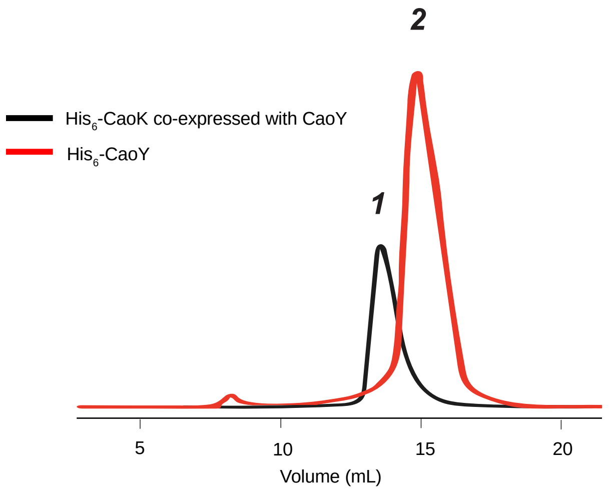

Figure S1. SEC of Ni-NTA IMAC-purified products from *E. coli* containing pRSFDuet-1-CaoY(MCSI)-His$_6$-CaoK(MCSII) (black), and pRSFDuet-His$_6$-CaoY(MCSI) (red).

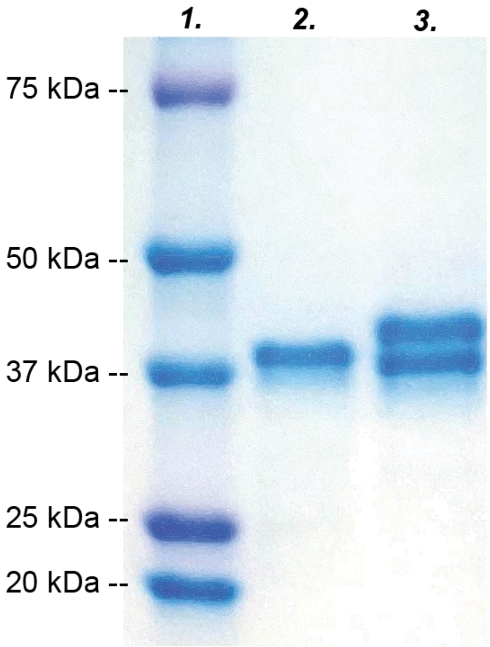

Figure S2. SDS-PAGE of SEC peaks shown in Figure S1. lane 1: protein ladder, lane 2: peak 2, lane 3: peak 1.

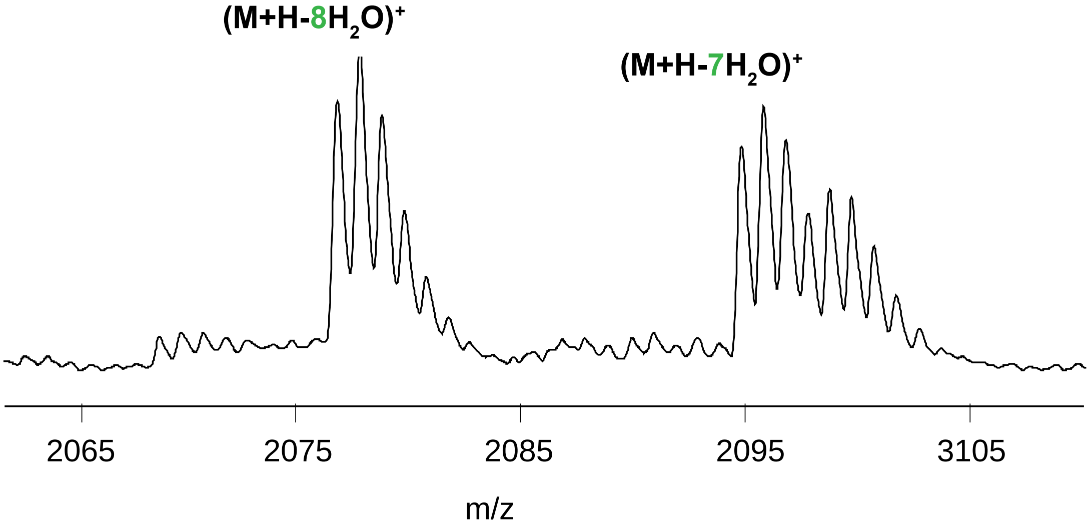

Figure S3. MALDI-TOF MS of Glu-C digested His_6-CaoA modified by CaoK and CaoY by co-expression in *E. coli*. $(M + H - 8\ H_2O)^+$ exp. m/z = 2075.9, obs. m/z= 2076.7. $(M + H - 7\ H_2O)^+$ exp. m/z = 2093.9, obs. m/z = 2094.6

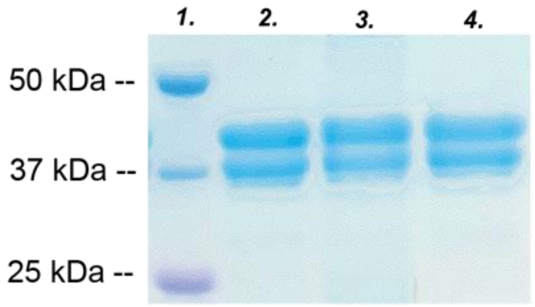

Figure S4. SDS-PAGE analysis of SEC fractions from Ni-NTA IMAC-purified proteins expressed in *E. coli*. lane 1: protein ladder, lane 2: expression of pRSFDuet-1-CaoY-R187A(MCSI)-His_6-CaoK(MCSII), lane 3: expression of pRSFDuet-1-CaoY-K219A(MCSI)-His_6-CaoK(MCSII), lane 4: expression of pRSFDuet-1-CaoY(MCSI)-His_6-CaoK(MCSII).

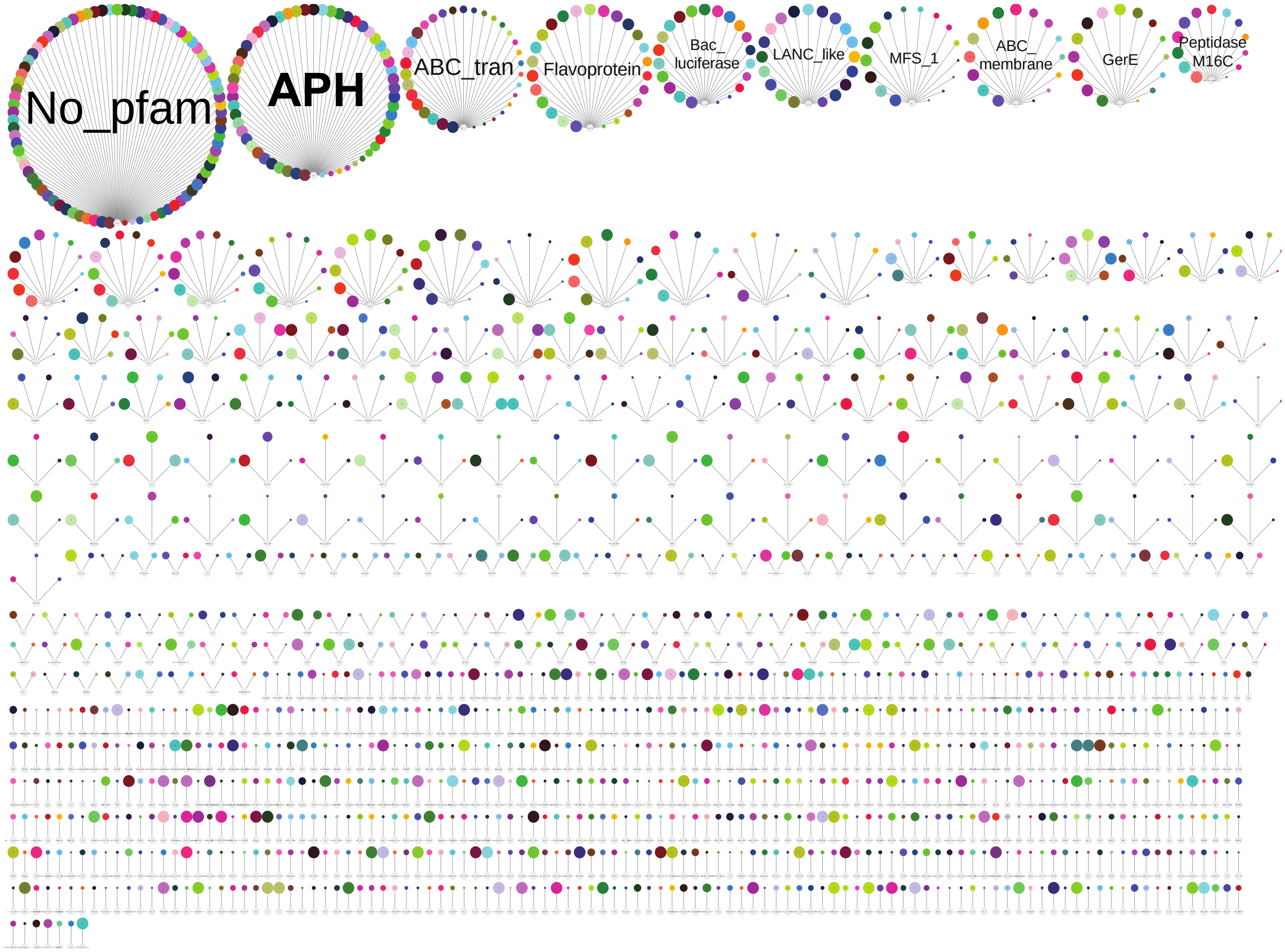

Figure S5. Pfam-based genome neighborhood network of CaoY homologues (PF17914) generated by the Enzyme Function Initiative-Enzyme Similarity Tool.[7, 8] In this analysis, 1,291 BGCs were retrieved from UniProtKB in total. Each "spoke-wheel" indicates a protein family that was co-occurring with the query (PF17914) with the co-occurring protein family labeled in the center of the circle. In each wheel, each node on the periphery with a specific color represents a group of similar BGCs that contains PF17914 proteins and the protein indicated in the center of the wheel. The size of the colored node is representative of the number of BGCs within the group. The most frequent proteins co-occurring with CaoY are members of the APH family proteins.

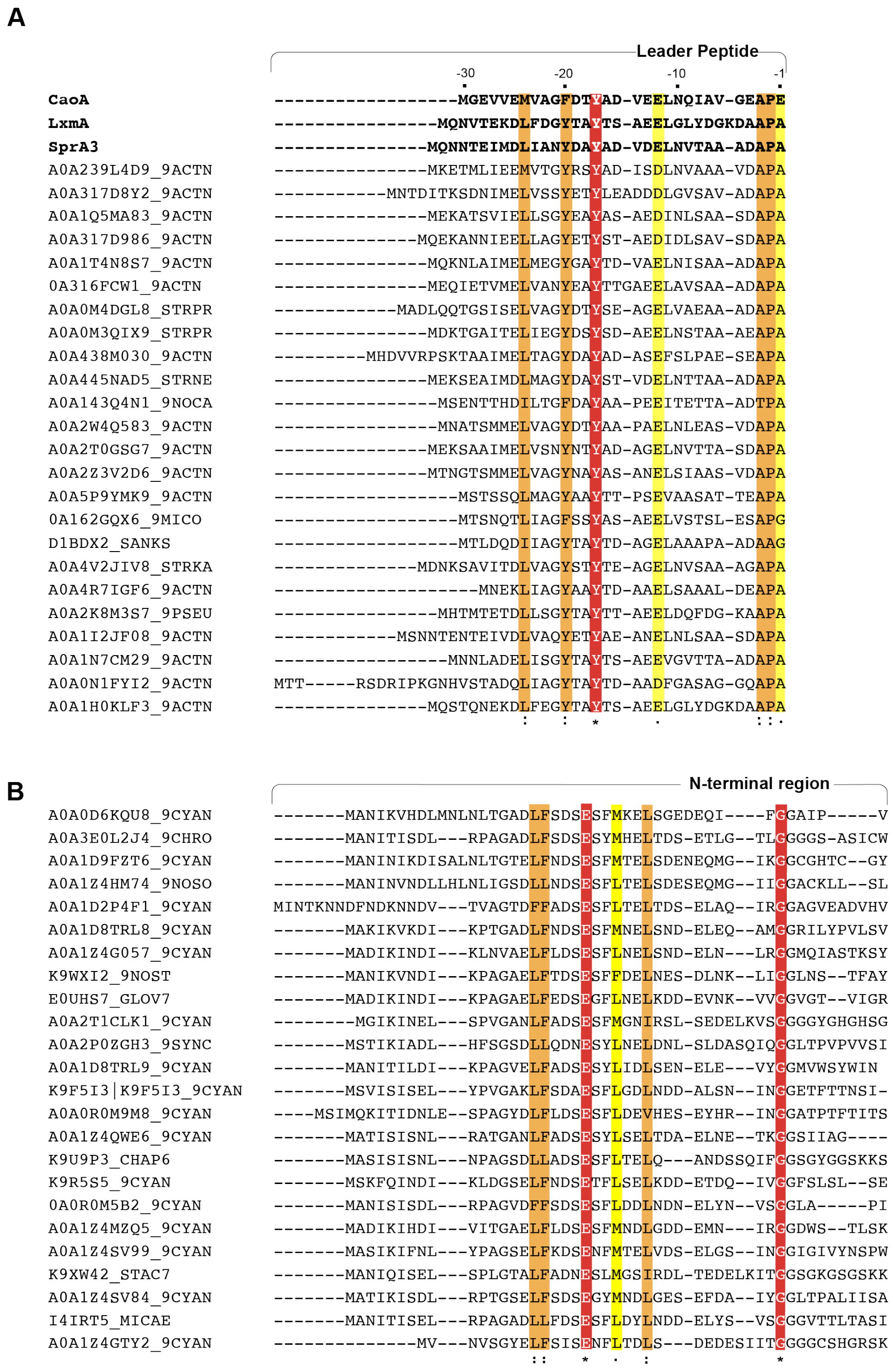

Figure S6. Multiple sequence alignment (MSA) of N-terminal sequences of select putative class V LanA precursor peptides that co-occur with the CaoKY pair retrieved using the GNN tool as described above. Further analysis identified two types of peptides shown in panels A and B that show similarity of leader peptides within each

group and that were termed type-A and type-B. In panel A, the N-terminal leader peptide sequences are aligned and positioned based on CaoA residue number; since the structure of cacaoidin is known, the end of the leader peptide is also known. The core sequences of these peptides also have sequence similarity. In panel B, the N-terminal putative leader peptide sequences are aligned; since no members have been characterized to date, it is not known where the end of the leader peptide is located. The core peptides of type B substrates are diverse. The UniProtKB ID of each peptide is depicted in the left column. Fully conserved residues are highlighted in red and highly conserved residues are in orange. Other conserved residues are in yellow.

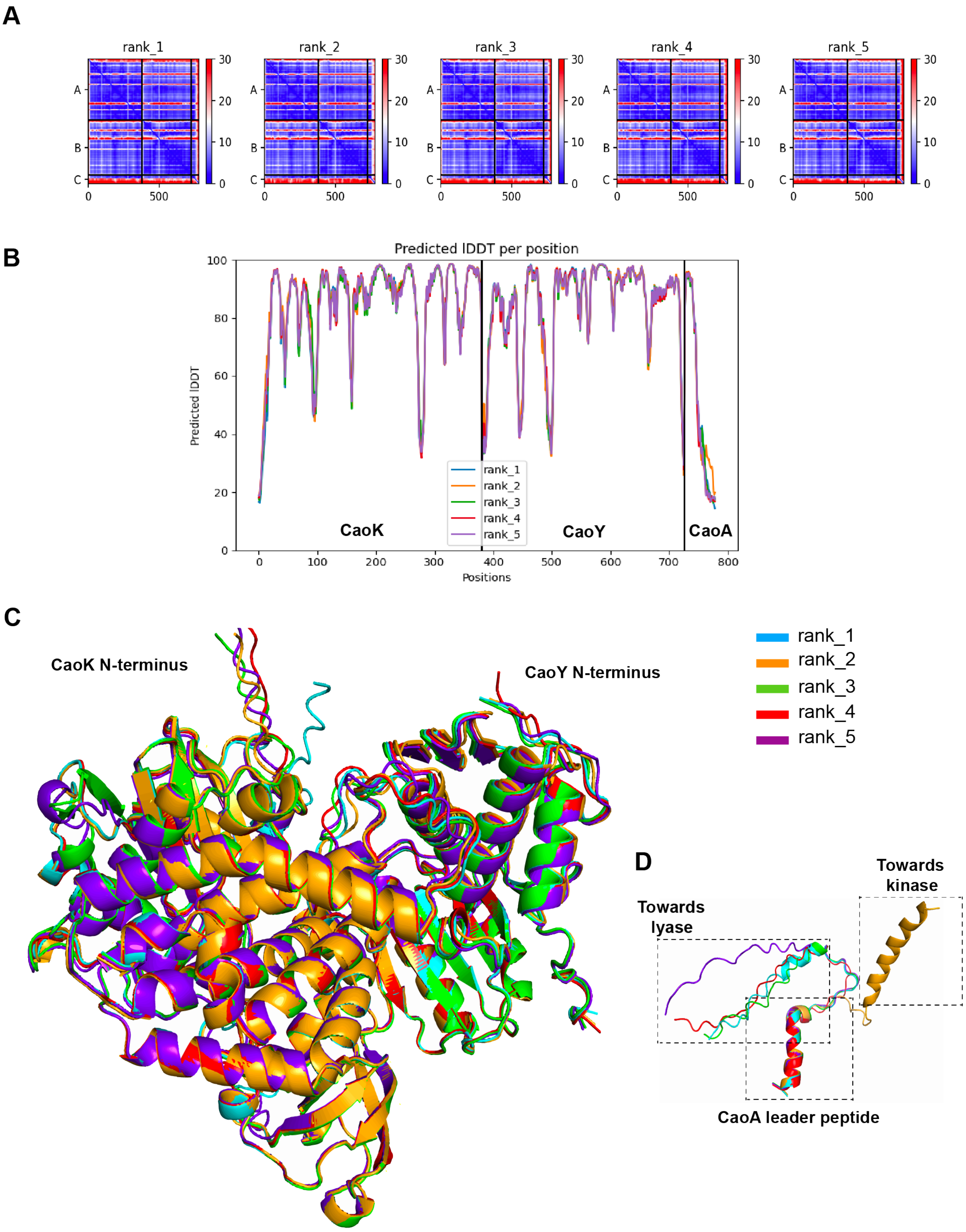

Figure S7. (A) The calculated Predicted Aligned Error (PAE) of the highest-ranking five models of CaoAKY predicted structures, from rank_1 to rank_5. The sequences were input into AlphaFold-Multimer in the order of A (CaoK), B (CaoY) and C (CaoA), which

are shown on the Y-axis. The residues from the three proteins are in this order shown on the X-axis and separated by vertical black lines. The color scale of PAE is shown on the right side of each figure, with blue representing PAE=0 and red PAE=30. (B) Per-residue confidence values (pLDDT) of the five models.[9] The sequences were input into AlphaFold-Multimer in the order of CaoK-CaoY-CaoA and the residues are positioned in this order on the X-axis of the figure. The vertical lines delineate CaoK, CaoY and CaoA. (C) Alignment of CaoK and CaoY from the five models. (D) Alignment of CaoA from the five models. The N-terminus of CaoA with high pLDDT values (Table S5) shows consistency for the five models. In contrast, the core peptide of the five models is poorly defined and in some models is oriented towards the lyase active site and in some models towards the kinase active site. This is not unexpected based on the way AlphaFold Multimer works and for enzymes that iteratively act on different parts of a substrate peptide.

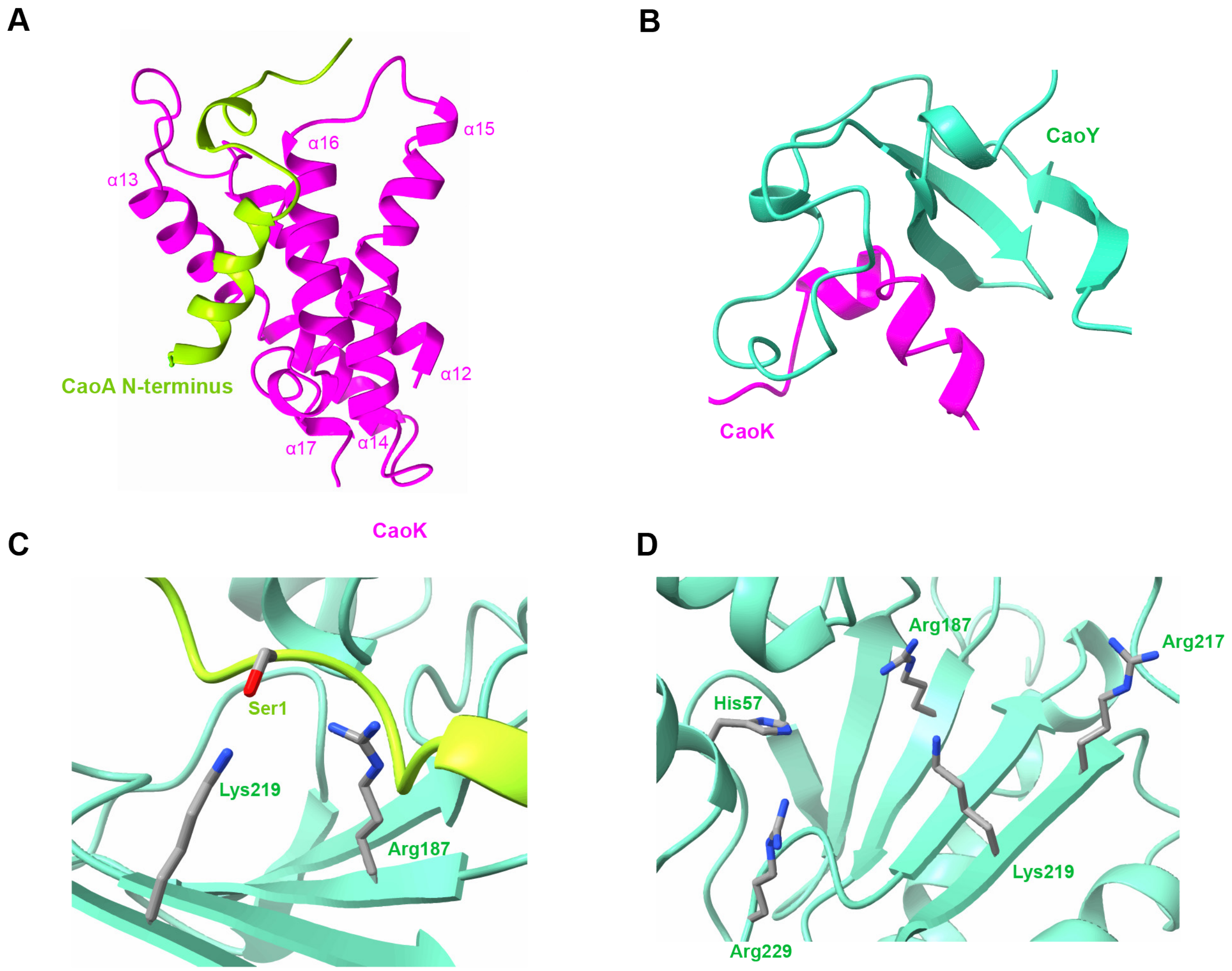

Figure S8. (A) CaoK C-terminal α-helices and the CaoA leader peptide in the rank_1 model. (B) CaoK-CaoY potential interaction region in the rank_1 model. (C) CaoY active site in the rank_1 model, with Arg187 and Lys219 from CaoY and Ser1 from CaoA depicted. (D) CaoY active site in the rank_1 model, with positively charged residues depicted. CaoK, CaoY and CaoA are colored in magenta, cyan and green, respectively.

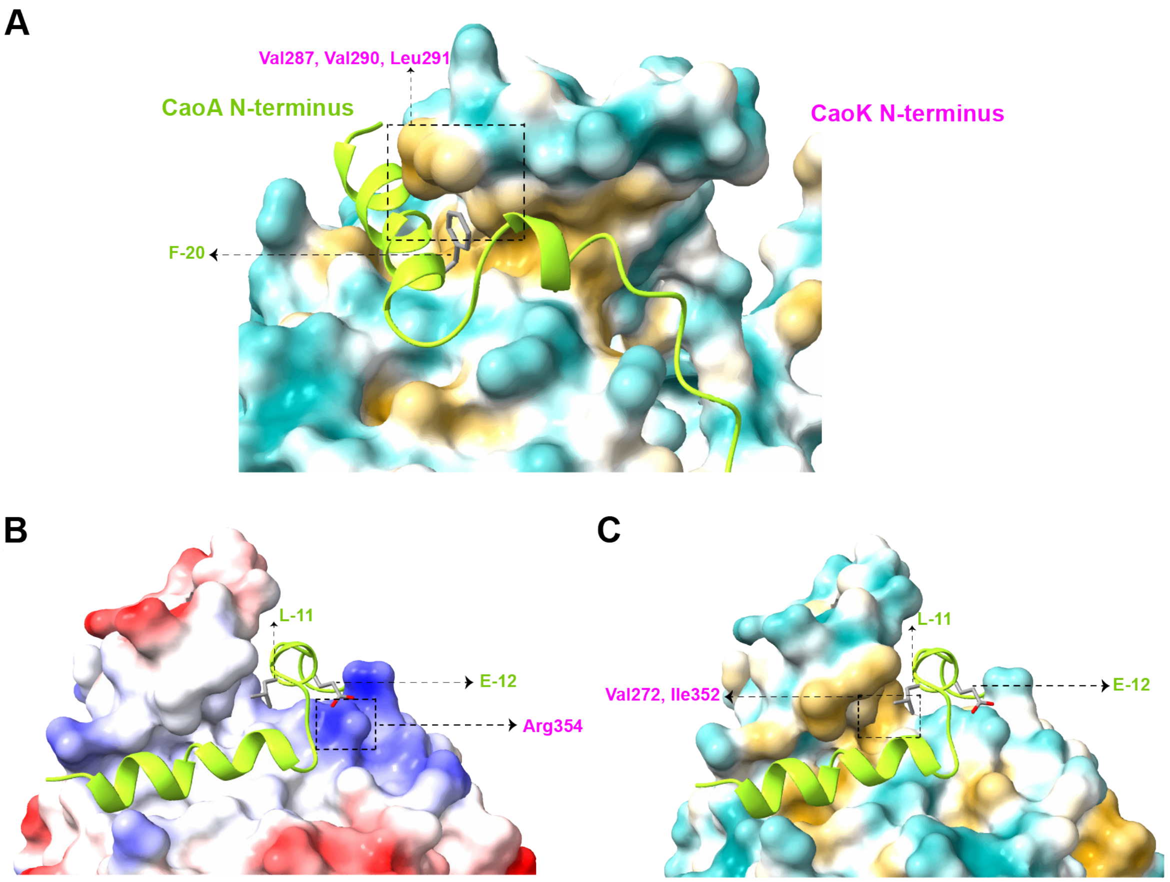

Figure S9. Potential interactions between the CaoA LP and the CaoK hydrophobic groove in the rank_1 predicted structure model. Phe−20 in CaoA LP and the predicted corresponding interaction site in CaoK are depicted in (A), and Glu−12 and Leu−11 in the CaoA LP and the corresponding predicted interaction sites in CaoK are depicted in (B) and (C), respectively. CaoA is colored in green, with CaoK hydrophobic surface in yellow and hydrophilic surface in blue in (A) and (C), or with CaoK negatively charged surface in red and positively charged surface in navy in (B). It should be noted that AlphaFold is less reliable for predicting side chain conformations and that these predictions may not be accurate.

E-28K

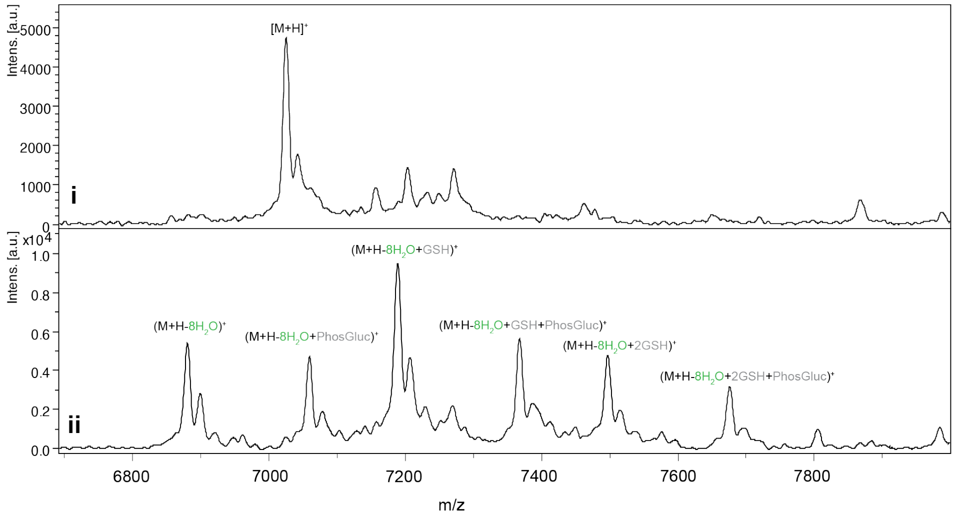

V-27T

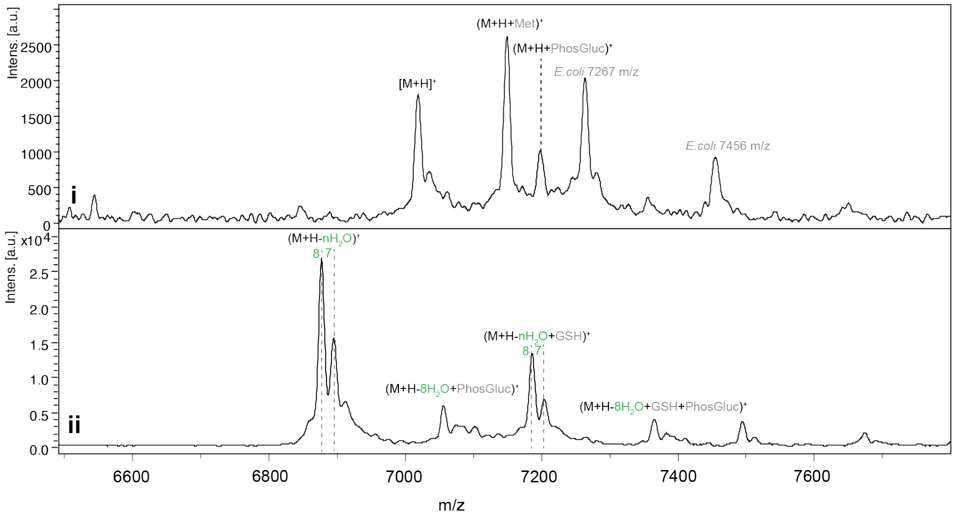

V-26T

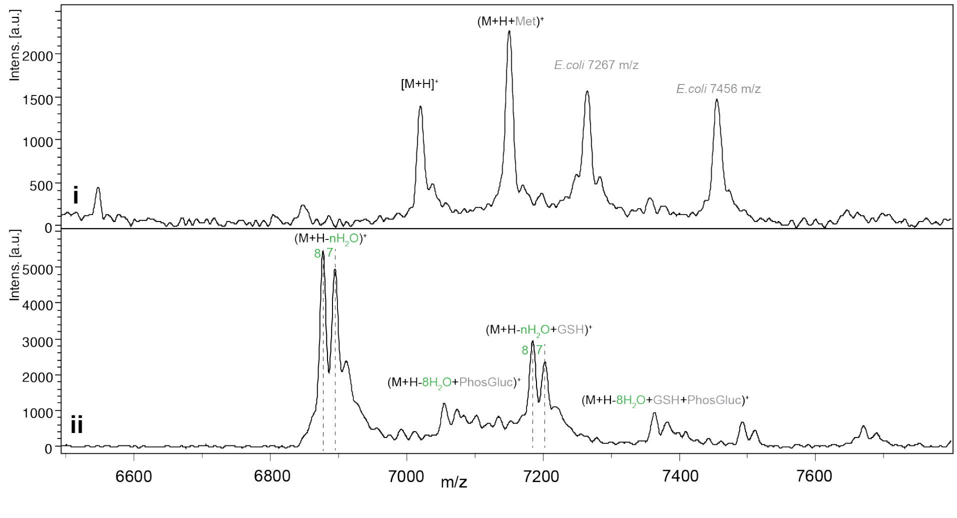

E-25K

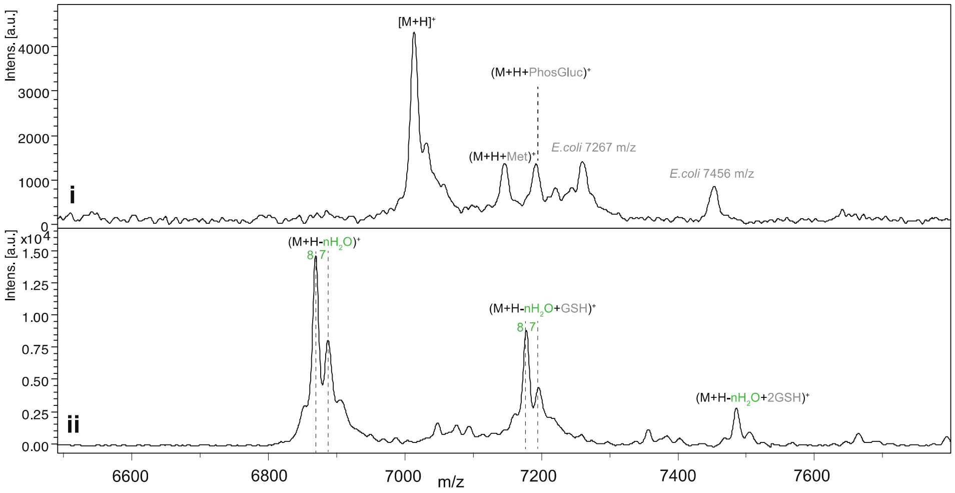

M-24Q

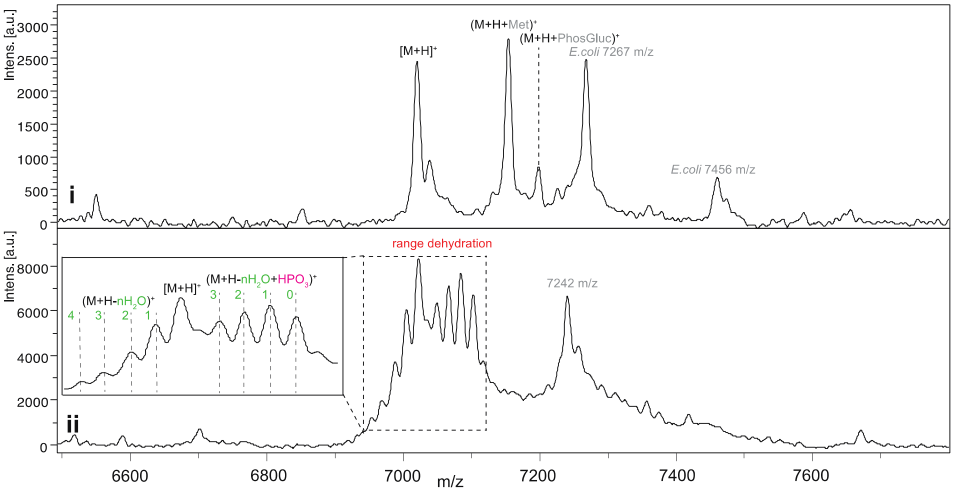

V-23T

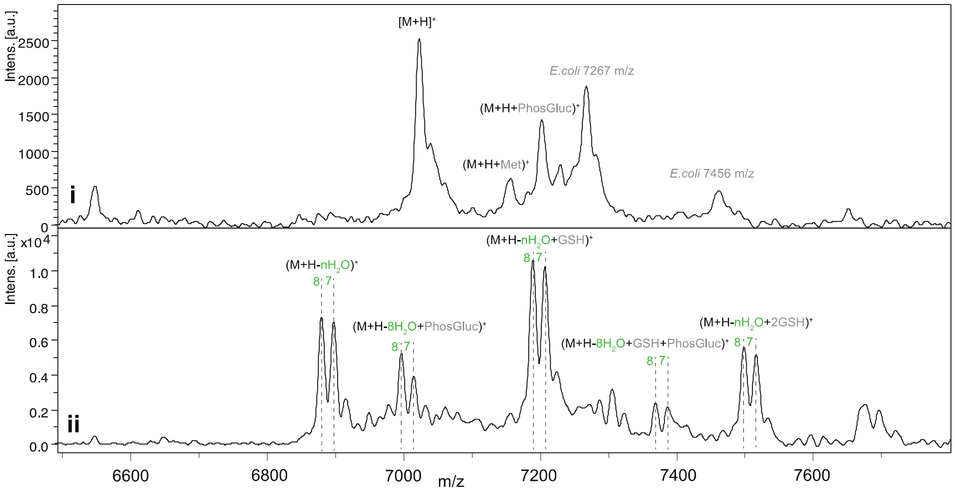

A-22S

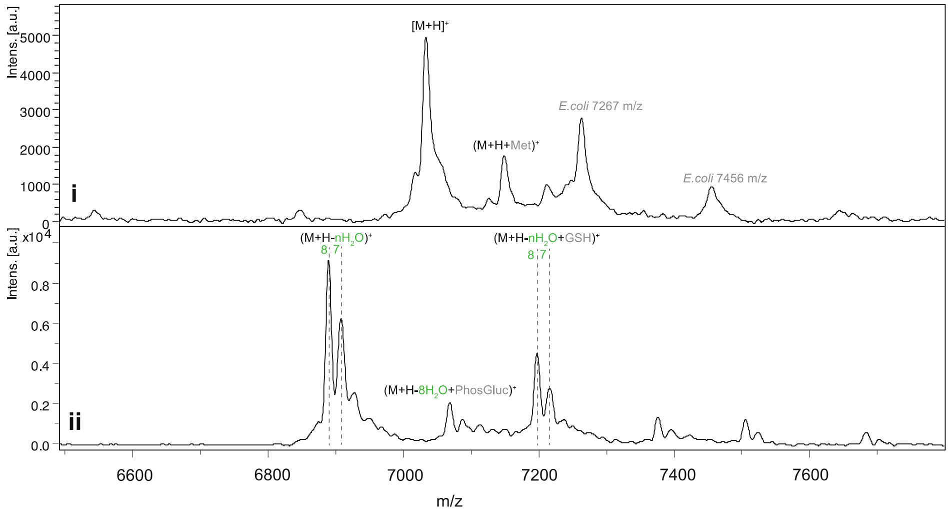

G-21S

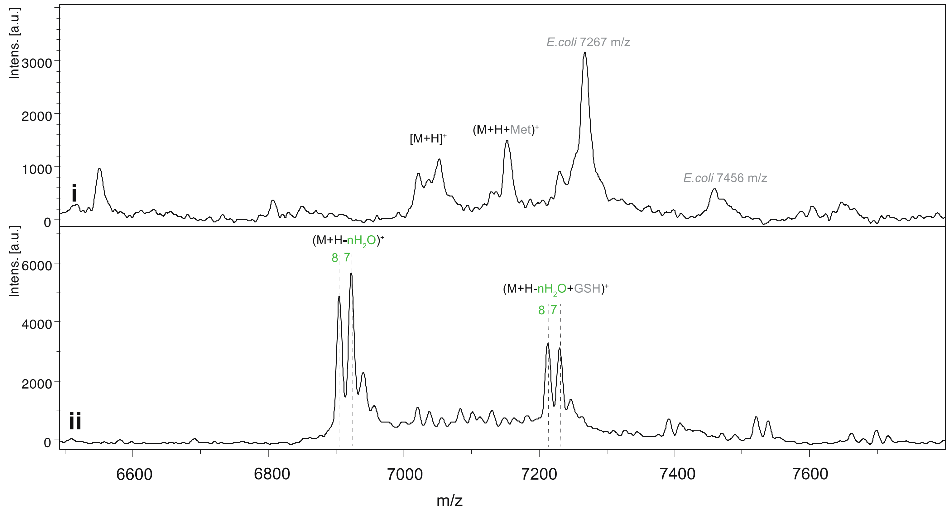

F-20Q

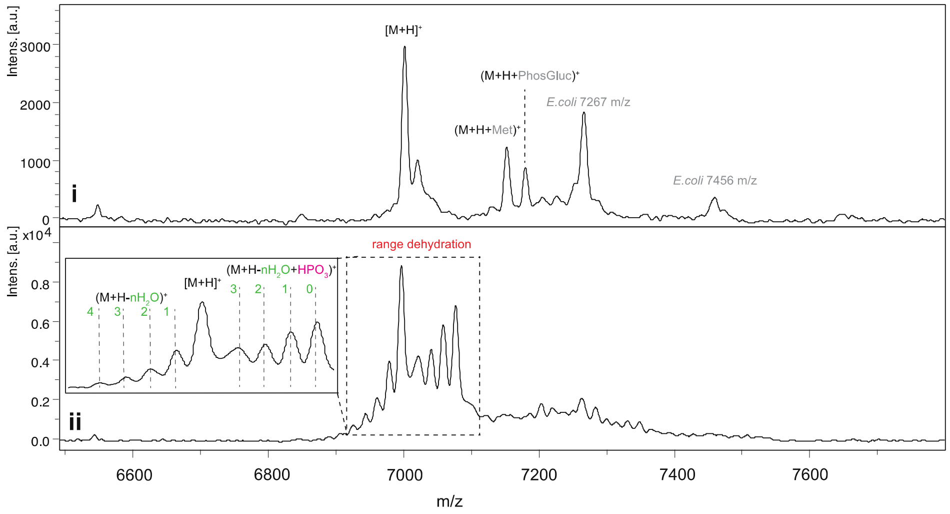

T-18A

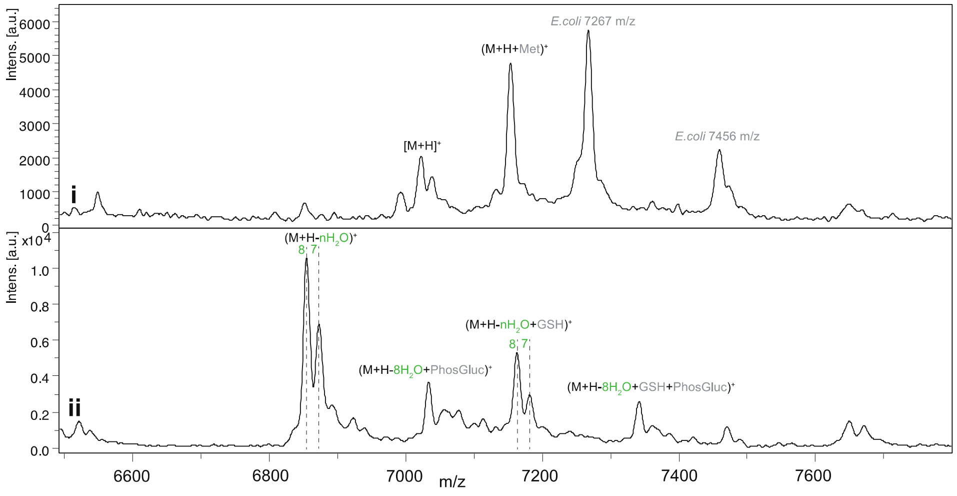

Y-17A

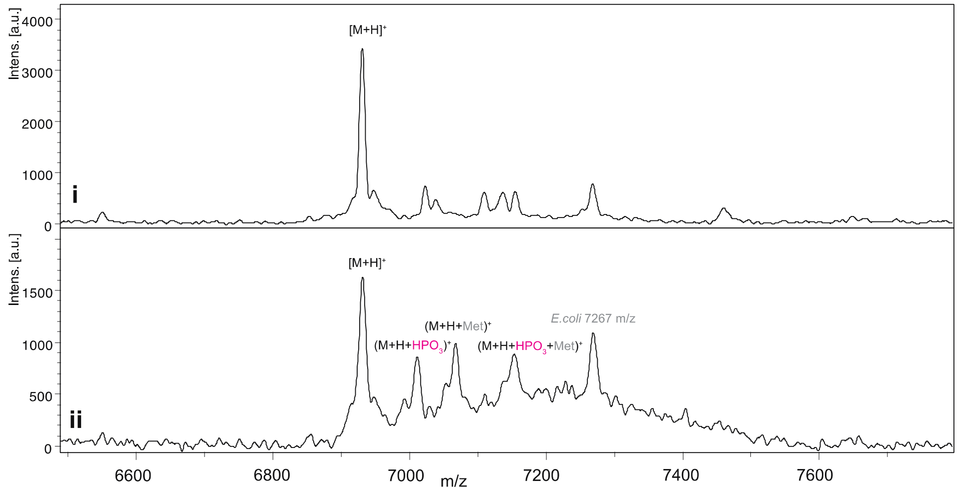

Y-17Q

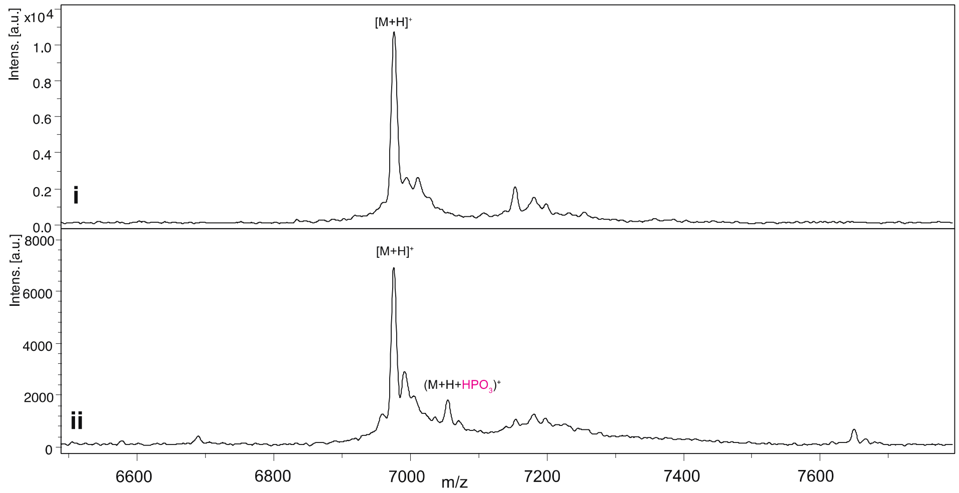

V-14T

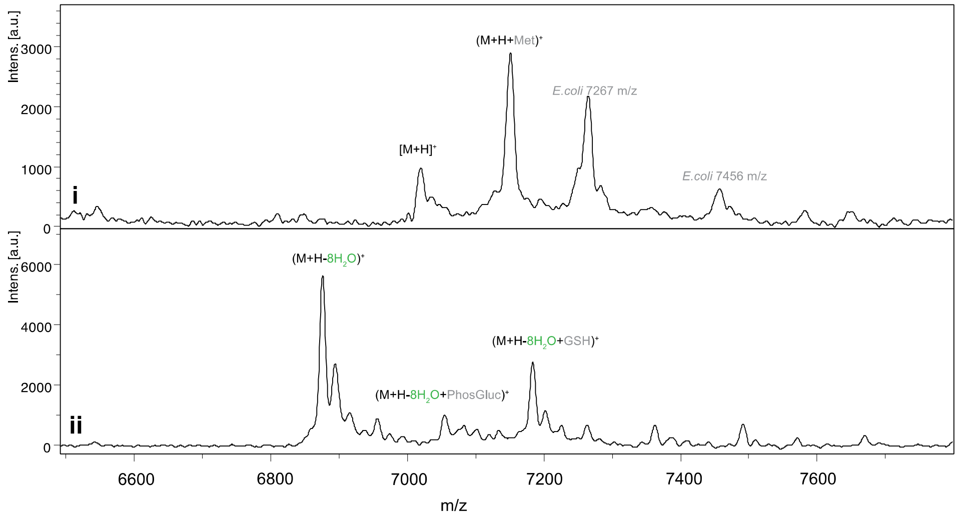

E-13K

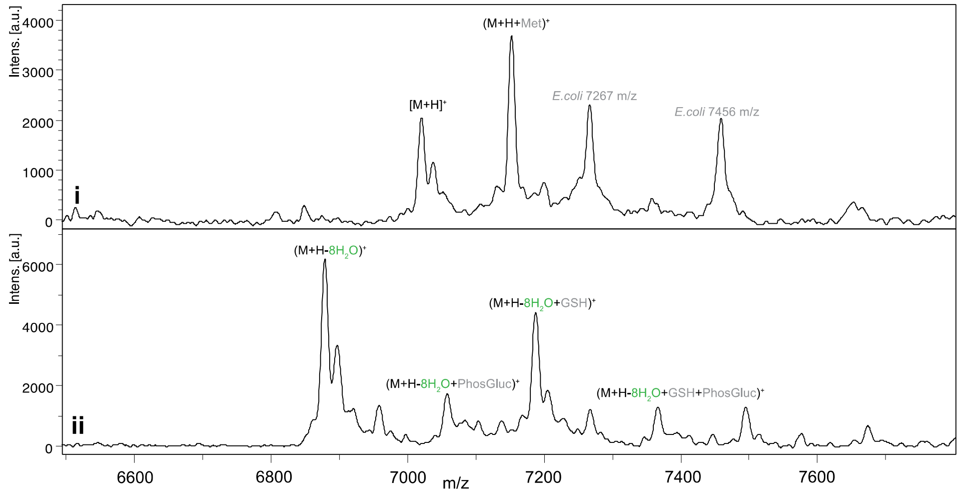

E-12K

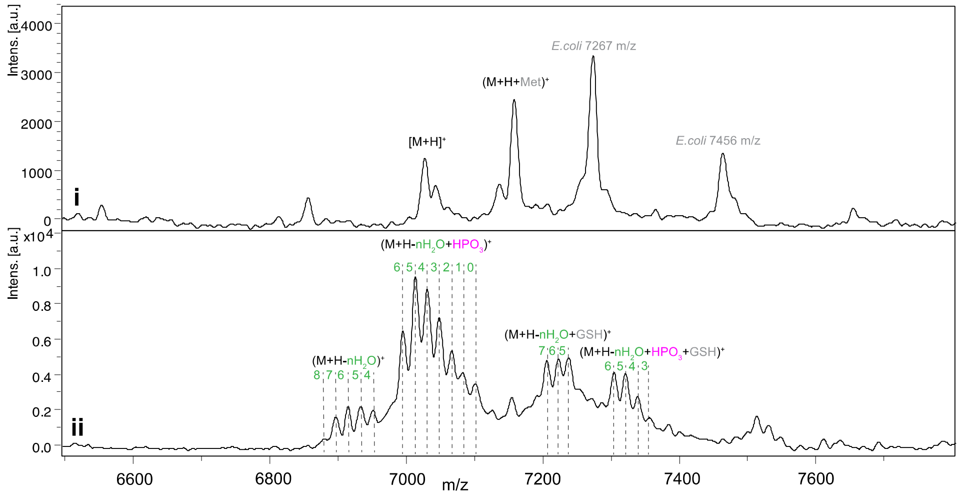

L-11N

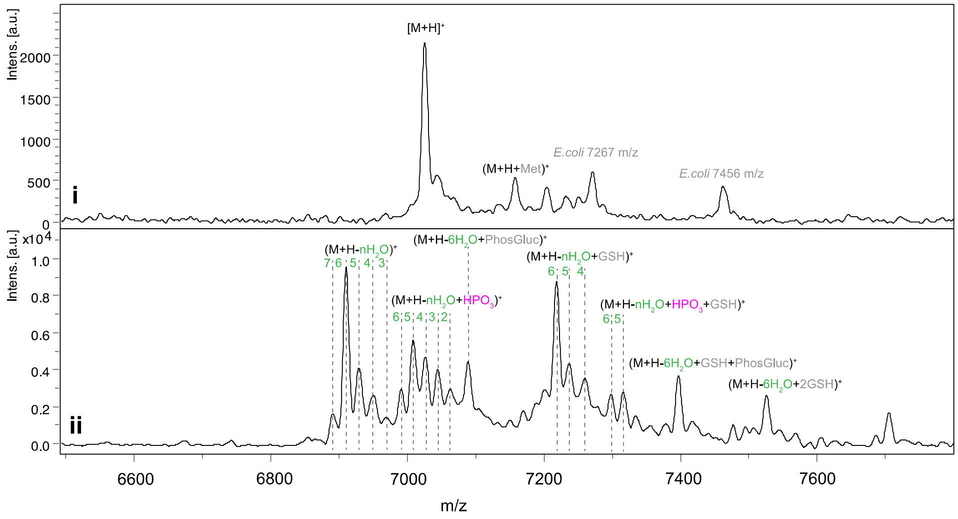

N-10L

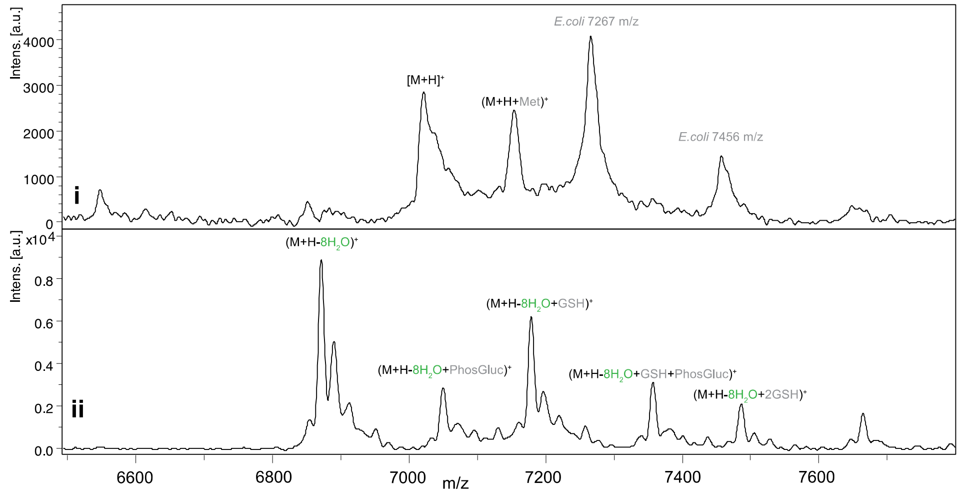

Q-9K

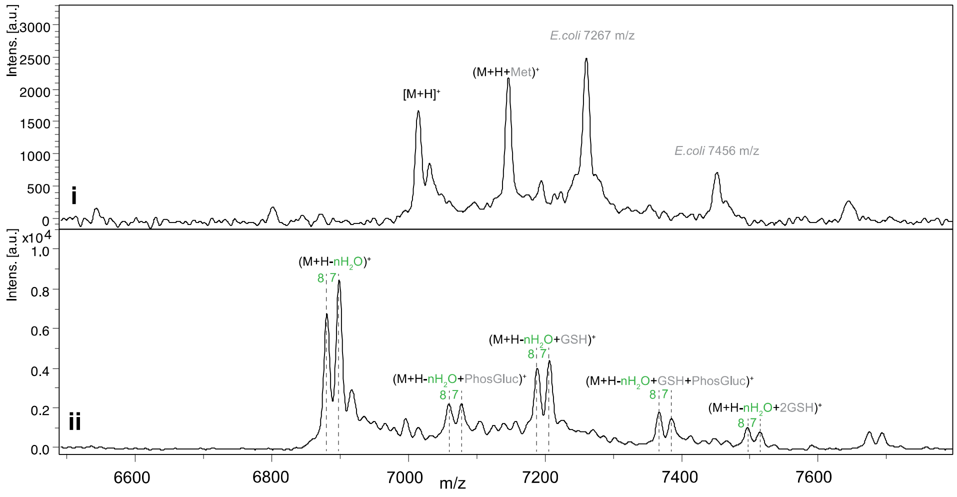

I-8K

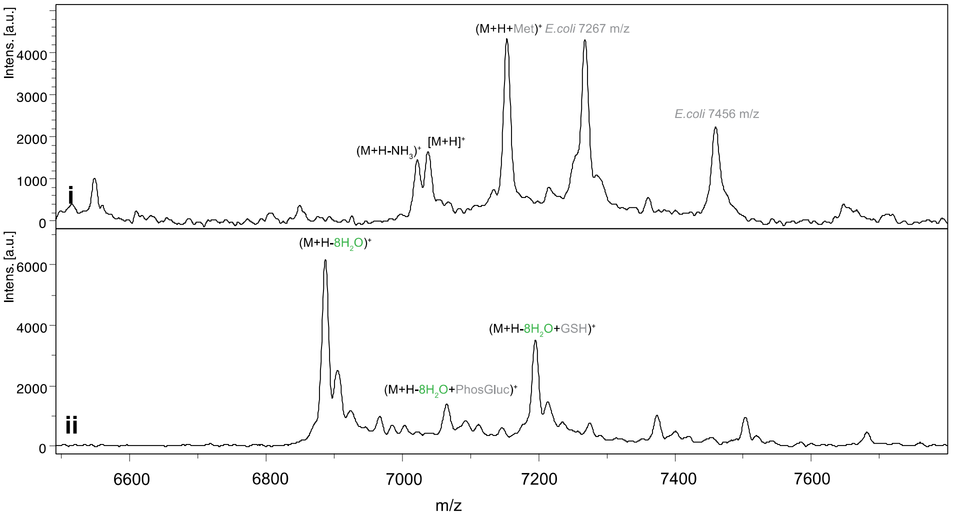

Intens. [a.u.]
(M+H+Met)+
E.coli 7267 m/z
E.coli 7456 m/z
(M+H-NH3)+
[M+H]+
i
(M+H-8H2O)+
(M+H-8H2O+GSH)+
(M+H-8H2O+PhosGluc)+
ii
m/z

V-6T

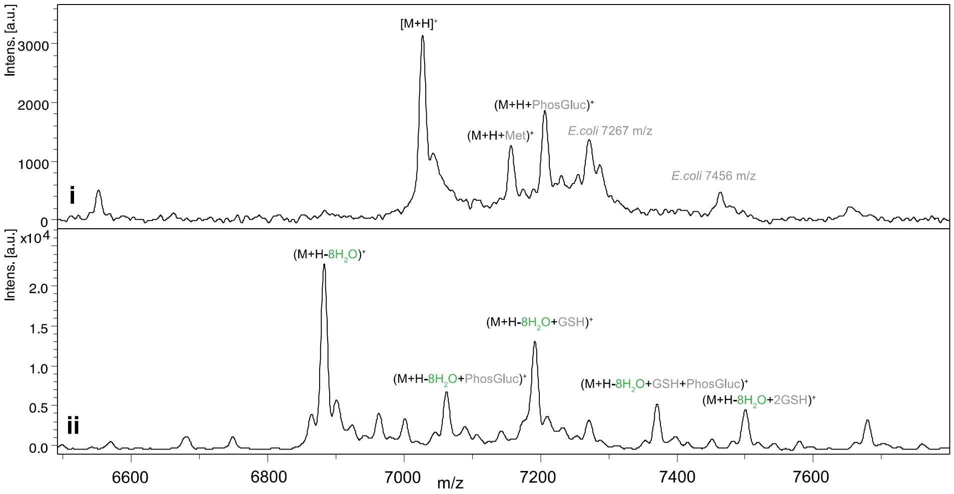

Intens. [a.u.]
[M+H]+
(M+H+PhosGluc)+
(M+H+Met)+
E.coli 7267 m/z
E.coli 7456 m/z
i
x10^4
(M+H-8H2O)+
(M+H-8H2O+GSH)+
(M+H-8H2O+PhosGluc)+
(M+H-8H2O+GSH+PhosGluc)+
(M+H-8H2O+2GSH)+
ii
m/z

E-4K

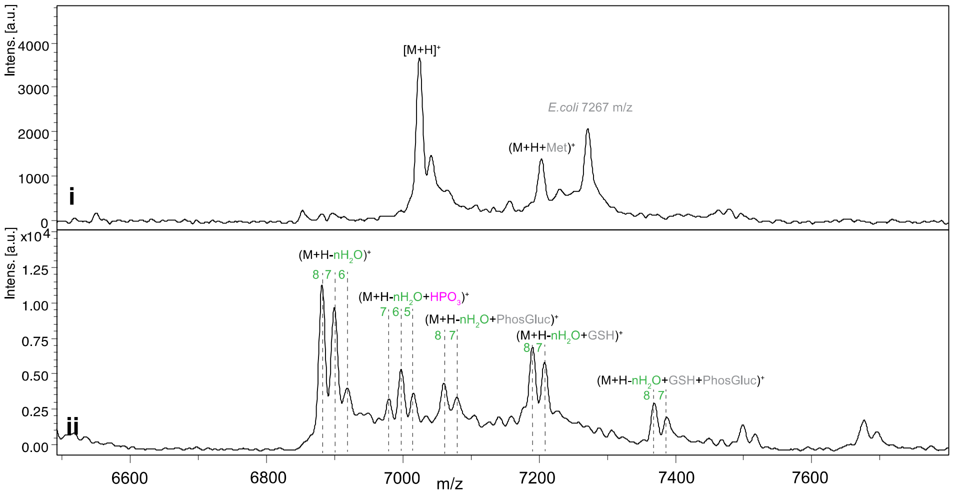

Intens. [a.u.]
[M+H]+
E.coli 7267 m/z
(M+H+Met)+
i
x10^4
(M+H-nH2O)+
(M+H-nH2O+HPO3)+
(M+H-nH2O+PhosGluc)+
(M+H-nH2O+GSH)+
(M+H-nH2O+GSH+PhosGluc)+
ii
m/z

A-3S

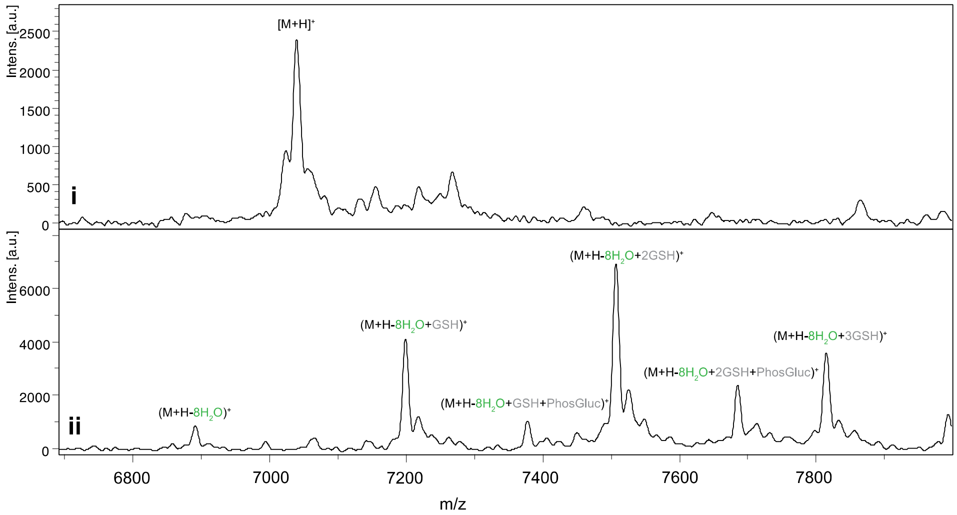
Intens. [a.u.]
[M+H]+
i
ii
(M+H-8H2O)+
(M+H-8H2O+GSH)+
(M+H-8H2O+GSH+PhosGluc)+
(M+H-8H2O+2GSH)+
(M+H-8H2O+2GSH+PhosGluc)+
(M+H-8H2O+3GSH)+
m/z

P-2A

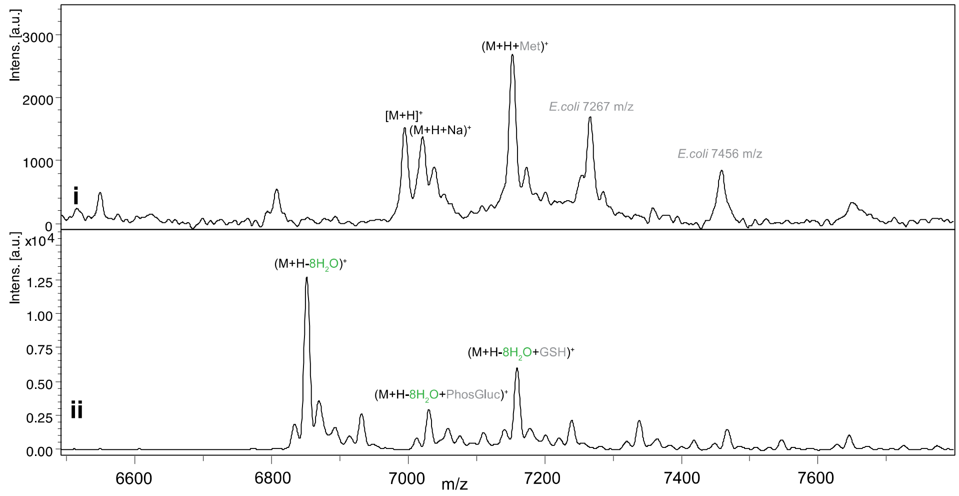
Intens. [a.u.]
[M+H]+
(M+H+Na)+
(M+H+Met)+
E.coli 7267 m/z
E.coli 7456 m/z
i
x10^4
(M+H-8H2O)+
(M+H-8H2O+PhosGluc)+
(M+H-8H2O+GSH)+
ii
m/z

E-1K

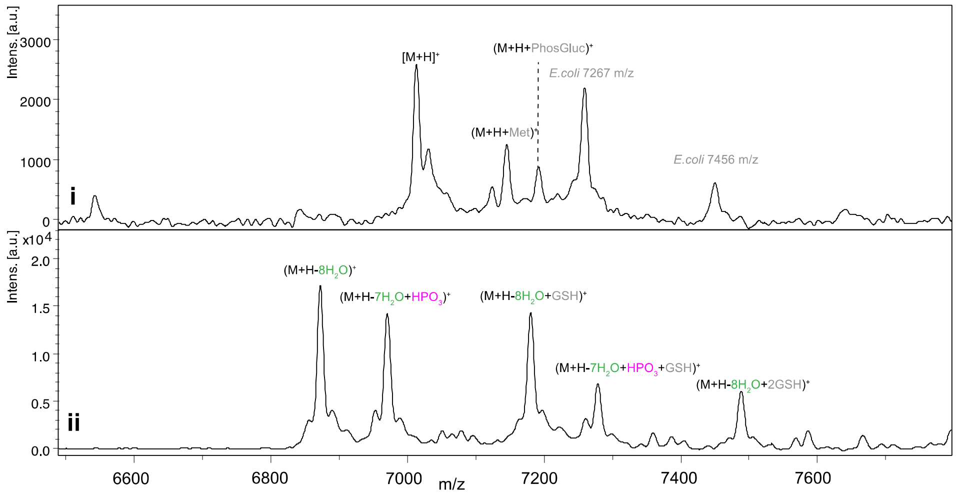
Intens. [a.u.]
[M+H]+
(M+H+PhosGluc)+
E.coli 7267 m/z
(M+H+Met)+
E.coli 7456 m/z
i
x10^4
(M+H-8H2O)+
(M+H-7H2O+HPO3)+
(M+H-8H2O+GSH)+
(M+H-7H2O+HPO3+GSH)+
(M+H-8H2O+2GSH)+
ii
m/z

Figure S10. MALDI-TOF mass spectra of full length His_6-CaoA variants expressed alone (i) and co-expressed with CaoK-CaoY (ii) in *E. coli*. +GSH: glutathionation, m/z = +305; +PhosGluc: *α*-*N*-Gluconoylation on the His_6 tag, m/z = +178; +Met: Methionyl, m/z = +131.

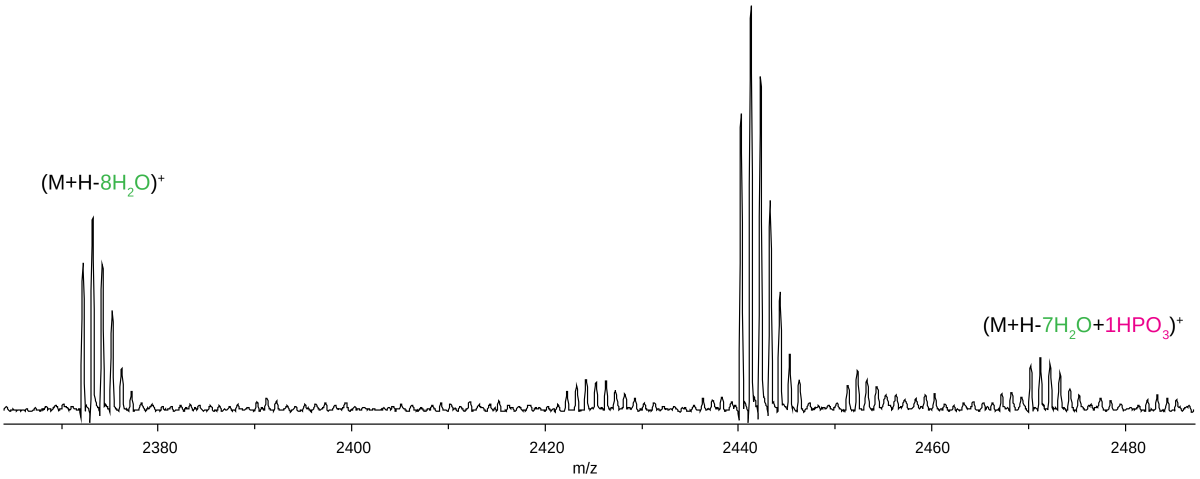

Figure S11. MALDI-TOF MS of Glu-C digested His6-CaoA-E−1K variant modified by CaoK and CaoY during co-expression in *E. coli*. $(M + H – 8\ H_2O)^+$ exp. m/z = 2372, obs. m/z= 2372; $(M + H – 7\ H_2O + HPO_3)^+$ exp. m/z = 2470, obs. m/z = 2470.

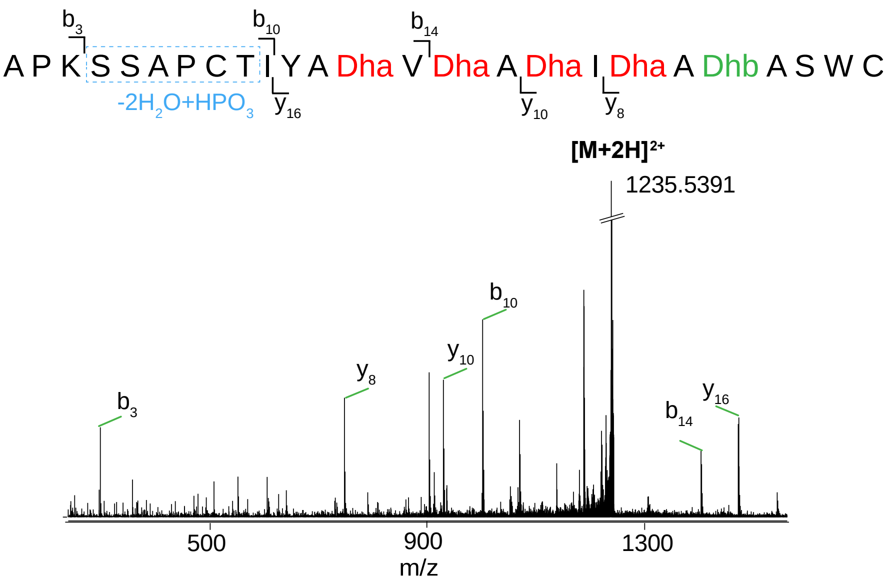

Figure S12. LC-ESI-QTOF MS-MS fragmentation pattern of Glu-C digested His6-CaoA-E−1K variant core peptide modified by CaoK-CaoY during co-expression in *E. coli*. For masses see Table S3.

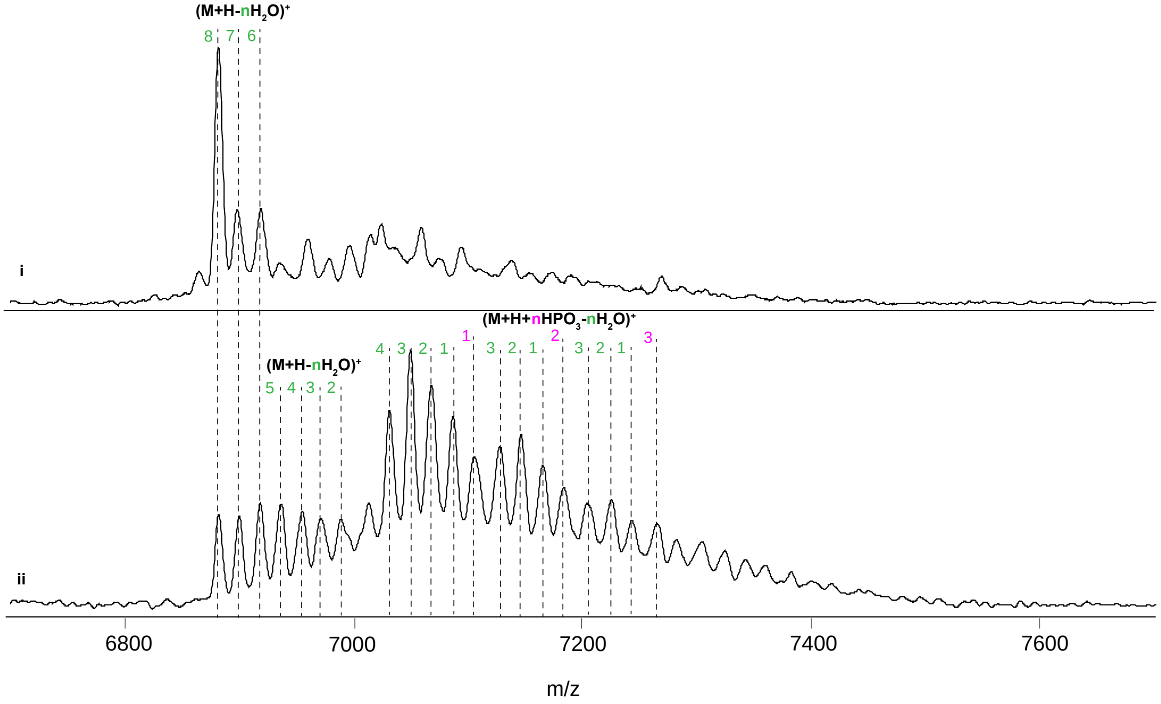

Figure S13. MALDI-TOF mass spectrum of (i) His$_6$-CaoA modified by CaoK-CaoY-R217Q in the presence of ATP and $MgCl_2$. $(M+H-8H_2O)^+$ exp.= 6881, obs.= 6880; $(M+H-7H_2O)^+$ exp.= 6899, obs.= 6896. $(M+H-6H_2O)^+$ exp.= 6917, obs.= 6917. (ii) His$_6$-CaoA modified by CaoK–CaoY-H57N in the presence of ATP and $MgCl_2$. $(M+H-8H_2O)^+$ exp.= 6881, obs.= 6880; $(M+H-7H_2O)^+$ exp.= 6899, obs.= 6898; $(M+H+HPO_3-3H_2O)^+$ exp.= 7051, obs.=7048; $(M+H+2HPO_3-2H_2O)^+$ exp.= 7149, obs.=7145.

References

1. Mirdita, M.; Schütze, K.; Moriwaki, Y.; Heo, L.; Ovchinnikov, S.; Steinegger, M., ColabFold - Making protein folding accessible to all. *bioRxiv* **2021**, 2021.08.15.456425.
2. Evans, R.; O'Neill, M.; Pritzel, A.; Antropova, N.; Senior, A.; Green, T.; Žídek, A.; Bates, R.; Blackwell, S.; Yim, J.; Ronneberger, O.; Bodenstein, S.; Zielinski, M.; Bridgland, A.; Potapenko, A.; Cowie, A.; Tunyasuvunakool, K.; Jain, R.; Clancy, E.; Kohli, P.; Jumper, J.; Hassabis, D., Protein complex prediction with AlphaFold-Multimer. *bioRxiv* **2021**, 2021.10.04.463034.
3. Mirdita, M.; Steinegger, M.; Soding, J., MMseqs2 desktop and local web server app for fast, interactive sequence searches. *Bioinformatics* **2019,** *35* (16), 2856-2858.
4. Mirdita, M.; von den Driesch, L.; Galiez, C.; Martin, M. J.; Soding, J.; Steinegger, M., Uniclust databases of clustered and deeply annotated protein sequences and alignments. *Nucleic Acids Res.* **2017,** *45* (D1), D170-D176.
5. Steinegger, M.; Meier, M.; Mirdita, M.; Vohringer, H.; Haunsberger, S. J.; Soding, J., HH-suite3 for fast remote homology detection and deep protein annotation. *BMC Bioinformatics* **2019,** *20* (1), 473.
6. Jumper, J.; Evans, R.; Pritzel, A.; Green, T.; Figurnov, M.; Ronneberger, O.; Tunyasuvunakool, K.; Bates, R.; Žídek, A.; Potapenko, A.; Bridgland, A.; Meyer, C.; Kohl, S. A. A.; Ballard, A. J.; Cowie, A.; Romera-Paredes, B.; Nikolov, S.; Jain, R.; Adler, J.; Back, T.; Petersen, S.; Reiman, D.; Clancy, E.; Zielinski, M.; Steinegger, M.; Pacholska, M.; Berghammer, T.; Bodenstein, S.; Silver, D.; Vinyals, O.; Senior, A. W.; Kavukcuoglu, K.; Kohli, P.; Hassabis, D., Highly accurate protein structure prediction with AlphaFold. *Nature* **2021,** *596* (7873), 583-589.
7. Zallot, R.; Oberg, N.; Gerlt, J. A., The EFI web resource for genomic enzymology tools: Leveraging protein, genome, and metagenome databases to discover novel enzymes and metabolic pathways. *Biochemistry* **2019,** *58* (41), 4169-4182.
8. Gerlt, J. A.; Bouvier, J. T.; Davidson, D. B.; Imker, H. J.; Sadkhin, B.; Slater, D. R.; Whalen, K. L., Enzyme Function Initiative-Enzyme Similarity Tool (EFI-EST): A web tool for generating protein sequence similarity networks. *Biochim. Biophys. Acta* **2015,** *1854* (8), 1019-37.
9. Mariani, V.; Biasini, M.; Barbato, A.; Schwede, T., lDDT: a local superposition-free score for comparing protein structures and models using distance difference tests. *Bioinformatics* **2013,** *29* (21), 2722-2728.
